# Supplementary material for: Longitudinal biomarker progression and validation for predicting operational tolerance in a prospective multicenter liver transplantation immunosuppression withdrawal trial
Source: PLoS One. 2025 Dec 8;20(12):e0326442. doi: 10.1371/journal.pone.0326442 (PMC12685220; doi:10.1371/journal.pone.0326442)
Supplement: S1 File — (DOCX) [file pone.0326442.s001.docx]

**Supplementary Methods**

**Eligibility criteria**

Patients aged between 18 and 75 years with LT were included if they met all of the following criteria: (a) liver transplant recipients with a normal functioning allograft and normal LFTs for over 3 years, (b) at least one year without a documented rejection episode, (c) no active neoplasia, following the criteria established in the consensus for the management of liver transplant patients with non-hepatic cancer[^1^](#_wqvcw1j64k1b)^,^[^2^](#_yvtxn176qmsh), nor a prior history of autoimmune liver disease before transplantation, (d) normal or minimal to focal mild portal mononuclear inflammation observed in a liver biopsy, or meeting the Banff Working Group criteria for immunosuppressant withdrawal, and (e) the ability to comprehend the informed consent. A baseline biopsy was conducted before the beginning of the study to validate the point “d” of the inclusion criteria. All liver biopsy analyses were centralized at HCUVA and performed by the same experienced pathologist in LT pathology.

**Immunosuppressive drug withdrawal protocol**

Patients who fulfilled the eligibility criteria were randomized in a ratio 2:1 to either immunosuppressive drug withdrawal (Study Group) or keeping conventional IS treatment (Control Group), respectively.

• Study Group (G1): In this group, patients underwent controlled complete withdrawal of IS over a period of 12 months. For patients on steroids and/or mycophenolate mofetil, these medications were discontinued during the first 6 months, while calcineurin inhibitors were gradually reduced in decrements of at least 10% at 1-month intervals over 12 months, as long as liver function remained stable from the beginning of IS withdrawal.

• Control Group (G2): In this group, patients maintained their baseline immunosuppression regimen as per standard clinical practice prior to randomization.

All patients in the current study belong to the G1 subgroup of the original clinical study, maintaining the sequence of patient selection, thus ensuring an unbiased sample.

**Patients follow up**

Patients were continuously monitored for liver graft dysfunction. Patients who did not develop rejection were classified as operationally tolerant as long as they maintained immunosuppressive drug cessation for at least 12 months and showed no histopathological evidence of acute and/or chronic rejection. Liver rejection was indicated by a persistent and significant increase in liver function tests and confirmed by liver biopsy. In cases where there was an increase in liver enzyme levels of less than three-fold, further reduction in IS dosage was withheld. Instead, a subsequent evaluation of liver function tests was scheduled within a 7 to 14-day timeframe. If the liver function tests returned to normal levels during this interval, a reduction in IS dosage was initiated according to the predefined protocol, followed by monthly monitoring. Conversely, in scenarios where liver function tests remained elevated by at least twice the baseline IS level, a progressive increment in IS dosage was undertaken until normalization of the tests was achieved. However, in cases where improvement was not observed or if the increase exceeded four-fold, a liver biopsy was performed to ascertain the presence of allograft rejection. In the event of a mild to moderate acute rejection diagnosis[^3^](#_jlfg8jbi684l), IS dosage was augmented until resolution of the rejection episode. Methylprednisolone boluses were administered when liver enzyme levels failed to normalize within a two-week period or in cases of severe acute rejection diagnosis[^3^](#_jlfg8jbi684l). Following the complete withdrawal of IS, a follow-up assessment was conducted at 6-week intervals for a span of 3 to 4 months. Subsequently, patients underwent reassessment at the outpatient clinic every 3 months. Absence of rejection was histologically confirmed 12 months after complete IS withdrawal.

The diagnosis of acute rejection followed the suggestions established by the Banff Working Group, based on the presence of predominantly mononuclear portal infiltrate plus nonsuppurative ductal cholangitis with or without endotheliitis^1^.

**Biomarker description**

These variables encompassed gene expression both in tissue [Hepcidine antimicrobial peptide (*HAMP)*, Suppressor of cytokine signaling 1 (*SOCS1)*, Transferrin receptor (*TFRC*)] and in peripheral blood [forkhead box P3 (*FOXP3)*, IKAROS family zinc finger 2 (*IKZF2)*, Fem-1 homolog C (*FEM1C)*, SUMO-specific peptidase 6 (*SENP6*)]. Additionally, we examined microRNA (miRNA) expression in peripheral blood (*miR31, miR95*), quantified the infiltration of CD4+FOXP3+ cells in liver tissue, and assessed of the degree of methylation within the *FOXP3* regulatory T cell specific demethylated region (TSDR) in both whole blood and tissue. Furthermore, we conducted an analysis of the expression of seven genes associated with subclinical inflammatory lesions[^4^](#_syvxjc2yegk1) in liver tissue. These genes included [Guanylate binding protein 2 (*GBP2*), Glycoprotein Nmb (*GPNMB*), Major histocompatibility complex, Class II, DM alpha (*HLA*-*DMA*), Hyaluronan mediated motility receptor (*HMMR*), Matrix metallopeptidase (*MMP*)*9*, *MMP7*, Phospholipase A2 group VII (*PLA2G7*)] (Table S1).

**Quantitative real time–polymerase chain reaction (qRT-PCR)**

Total RNA was extracted from stabilized whole blood using the PAXgene Blood miRNA Kit (PreAnalytiX), and miRNeasy FFPE kit (Qiagen, Hilden, Germany) for liver tissue, followed by reverse transcription. Quantitative PCR was performed using SYBR Premix ExTaq (Takara Bio, Kusatsu, Japan) for mRNA and the miRCURY LNA miRNA PCR assays (Qiagen) for miRNA. Primers specific to the mRNA and miRNA were purchased from Qiagen.

The samples were run in duplicate, and the relative gene expression levels were calculated using the 2^-ΔCt^ method, normalizing to GAPDH and HPRT for mRNA and RNU6B for miRNA.

**Determination of the degree of methylation of the *FOXP3*-TSDR by pyrosequencing**

Genomic DNA (gDNA) was extracted from buffy coats using the innuPREP Blood DNA Mini Kit (Analytik Jena, Jena, Germany), and Blood DNA FFPE kit for liver biopsies (Qiagen). The DNA was then bisulfite converted using the EZ DNA Methylation Kit (Zymo Research, Irvine, CA, USA), and the *FOXP3* TSDR methylation ratio (TSDR-MR) was determined by pyrosequencing in a PyroMark Q24 instrument (Qiagen). Method and primers design were as follow:

| **PCR forward primer (5' to 3')** | Btn-TATTTTATTTGGGTTAAGTTTGTTGTAGG |
| --- | --- |
| **PCR reverse primer (3' to 5')** | ATCTACATCTAAACCCTATTATCAC |
| **Sequencing primer (5' to 3')** | ACTTACCCAAATTTTTCC |
| **No. of CpG sites analyzed** | 8 |
| **PCR conditions** | 95°C_15 min  40 cycles 94°C_30s 50°C_30s 72°C_30s  72°C_10min  4°C_hold |
| **Sequence to analyze** | RCCATTAACRTCATAACRACCRAATACRCCRAACTTCATCRACACCACRAAAAAAAAAAAAAAAAC |
| **Nucleotide dispensation order** | CGACATGATCGATCATATCAGATCAGATGATCGATCAGACTCATACAGACACATCAAGAA |

**Immunohistochemistry**

To determine the CD4^+^FOXP3^+^ lymphocyte infiltrate in PaxGene-fixed and paraffin embedded liver samples, an indirect immunohistochemistry was carried out in 4µm-thick sections from the samples. Briefly, after deparaffination and rehydration the sections were underwent to an antigen retrieval procedure by using a commercial solution (Target retrieval solution High pH, K8004 (Agilent-Dako, Santa Clara, CA, USA) at 98ºC for 2º min (PT-link module, Thermo Scientific). After endogenous peroxidase blocking (blocking solution, S2023, Dako-Agilent) for 5 min at RT, sections were incubated in normal goat serum for 20 min at 37ºC (S1012-50, Vector Labs, Burlingame, USA) to background-blocking, and overnight with primary antibodies (Monoclonal Mouse anti-CD4, IR669, Agilent-Dako, Ready-to-use; Monoclonal rat anti-FOXP3, 14-5773-82, Thermo Scientific, dilution 1:100). After the incubacion with the secondary AP-labelled anti-mouse polymer (S30095, Vector Labs) and HRP-labelled anti-rat polymer (S30032, Vector labs), sections were finally revealed with DAB (dark-bown precipitated, K346711-2, Agilentg-Dako)) and AP (red, SK4605, Vector Labs) and hematoxylin-counterstained. Double-positive cells were identified by a dark-brown precipitated with nuclear pattern with a red precipitated with membrane-pattern.

Sections were then digitalized by using a high resolution slide scanner (Pannoramic MIDI-II, 3D Histech, Budapest, Hungary) and examined with a digital especialized software (Slide Viewer ver. 2.6.0.166179, 3D Histech). The infiltration of CD4^+^FOXP3^+^ lymphocyte in liver biopsies was finally determined by direct count of double-positive cells in 400X fields.

**Analyzing the Longitudinal Evolution of Markers**

To assess various aspects of the longitudinal evolution of markers, we employed Generalized Additive Mixed Regression Models (GAMM) as proposed by Wood in 2017[^5^](#_512l09wbowmq). GAMM models, which extend traditional linear regression models, serve as a valuable statistical tool in clinical research for exploring complex relationships between various predictors and clinical outcomes.

GAMM models are particularly beneficial when dealing with data that may exhibit non-linear patterns (i.e., the relationship between the outcome and predictor does not follow a linear trajectory) or when different individuals within the study may respond differently to the same interventions. These models go beyond conventional linear models by allowing for the inclusion of non-linear or smooth predictor effects, as well as interactions between factor variables and the smooth effects of predictors. In other words, they accommodate situations where the effect of the predictor on the response may not be linear and could vary among different levels of the factor.

Additionally, GAMM models can account for the potential correlation between repeated measurements on the same subjects, considering the longitudinal nature of the data, where each patient undergoes multiple measurements over time. The general form of a GAMM model can be expressed as follows:

$$Y_{ij}=\beta_{0}+\beta_{1}F_{ij}+s\left( X_{ij} \right)+Z_{i}b+\epsilon_{ij}$$

Here, $Y_{ij}$ represents the outcome for the *j*th observation within patient *i*, $\beta_{0}$ is the global intercept, $\beta_{1}F_{ij}$ is the slope for the factor variable *F* (or other parametric variable included in the model), $s_{X_{ij}}$ denotes the flexible/smooth effect of the predictor on the outcome, and $\epsilon_{ij}$ the error term for the *j*th observation within patient *i*. The term representing the random effects is $Z_{i}b$, being *b* the vector of random effects coefficients. Various types of random effects can be included in the model to represent individual patient-specific effects. Among these, the random intercept allows only the intercept to vary for each patient, and this random term is selected for the estimated models.

When an interaction between the smooth term and a factor is included in the model, the term representing this interaction could be written as $s\left( X_{ij} \right)F_{i}$.

Penalized splines were chosen to model the smooth effects within the GAMM. Penalized splines are a flexible statistical technique that enables the capture of flexible relationships between the predictor and the outcome, without assuming specific mathematical forms. These splines are particularly valuable when dealing with complex data patterns. The smoothness of the curve, indicating the degree of flexibility of the effect, is controlled through penalization to prevent overfitting and model instability. An essential concept to penalized splines is degrees of freedom (df), as this parameter controls the flexibility of the smooth function. Increasing the degrees of freedom results in a more flexible curve, whereas fewer degrees of freedom yield a smoother curve. The number of degrees of freedom is automatically selected using a technique known as Generalized Cross-Validation (GCV)^[5](#_512l09wbowmq)^, which finds the optimal balance between model flexibility and goodness of fit. This approach allowed us to effectively model the flexible associations in the clinical data while maintaining interpretability and avoiding unnecessary complexity.

GAMM models offer a flexible framework for clinical research to uncover relationships between factors of interest and clinical outcomes, thereby enhancing our understanding of disease mechanisms. For a more comprehensive overview of these models, please refer to Wood (2017)[^5^](#_512l09wbowmq).

The GAMM model selected for **evaluating the differences in marker values between Tol and non-Tol patients** at different time points had the form $Marker_{ij}=s\left( Timepoint_{ij} \right)+u_{i}+\epsilon_{ij}$ (parametric part not shown). We constrained the degrees of freedom to 1 to obtain a linear regression model, enabling the estimation of means for the marker at different time points, with patient ID included as a random intercept ($u_{i}$). After estimating the model, we obtained the least-squares means (also known as marginal means) and their 95% confidence intervals, allowing us to calculate the contrasts between these estimates at each time point (mean differences). The longitudinal nature of the data was considered when estimating standard errors and confidence intervals.

To assess **differences in the temporal evolution between Tol and non-Tol patients**, we selected a GAMM model of the following form: $Marker_{ij}=s\left( Time_{ij} \right)Group_{i}+u_{i}+\epsilon_{ij}$ (parametric part not shown). The term $s\left( Time_{ij} \right)Group_{i}$ represents the smooth effect of time, which can vary across the levels of the Group (Tol and non-Tol). After estimating the curves for each group, we obtained the differences between them through differentiation, along with the 95% confidence interval for this difference. The difference smooth represents the significant difference between the curves of the two groups when the smooth confidence interval does not include 0.

For estimating the **probability of tolerance based on marker level and follow-up time**, we employed a logistic GAMM of the following form: $P\left( Y_{ij}=Tolerance \right)=s\left( Marker_{ij} \right)Timepoint_{i}+u_{i}+\epsilon_{ij}$ (parametric part not shown). The term $s\left( Marker_{ij} \right)Timepoint_{i}$ represents the smooth effect of the marker value on the probability of tolerance, which can vary along time points.

## LASSO regularization method

In the process of constructing a predictive model to identify tolerant patients based on the “baseline" values of blood (B) and tissue (T) markers, variables were carefully selected using a regularization and selection technique known as LASSO (Least Absolute Shrinkage and Selection Operator)^[6](#_5sgh8e5woxh0)^ or L1 regularization. The primary goal of regularization methods is two-fold: to reduce overfitting and variance while mitigating the influence of less relevant predictors. LASSO regularization achieves this by identifying a subset of crucial predictors while effectively shrinking the coefficients of less relevant ones toward zero. This shrinkage is carried out by penalizing the sum of the absolute values of the regression coefficients using a tuning parameter denoted as lambda ($\lambda\sum_{j}^{p} \left| \beta_{j} \right|$). As lambda increases, a higher penalty is imposed on the regression coefficients, resulting in the exclusion of more predictors. The optimal value of lambda (*λ_min_*), which minimizes the mean cross-validated error, is determined through 10-fold cross-validation. This meticulous fine-tuning of the penalty parameter, lambda, aims to strike a delicate balance between model complexity and predictive accuracy, ensuring that the selected model remains both predictive and interpretable. In order to favor model parsimony and reduce potential overfitting, the largest lambda value within one standard error of the minimum error (λ 1-standard-error, *λ_1SE_)* was also used. Model performance and stability were assessed by calculating the mean and standard deviation of the cross-validation error at *λ_min_* to evaluate variability across folds. Furthermore, learning curves were constructed by training models on increasing subsets of data to compare training and validation area under the ROC curve (AUC), quantifying the performance gap as an indicator of potential overfitting. The final model was reported both at *λ_min_* and λ*_1SE_*, with emphasis on balancing predictive accuracy and model simplicity. The cross-validated error (mean ± 1 standard error) across the sequence of lambda values in the LASSO regression path, along with *λ_min_* and *λ_1SE_* estimated, are shown in Supplementary Figure 4.

For predictor variables with missing values, we employed the Predictive Mean Matching (PMM) method[^8^](#_vvcynkeo2e8b) to impute the missing data, ensuring that the missing pattern is not Missing Completely At Random (MCAR). PMM imputes missing values by matching them with observed values from the same or similar individuals based on the predictive distribution of the variable of interest. This imputation approach preserves the data structure and respects the clinical context (patient characteristics) while handling missing values, resulting in less biased estimates compared to other methods[^9^](#_7eceb7oby38w).

### **References**

1. Al-Adra DP, Hammel L, Roberts J, et al. Pretransplant solid organ malignancy and organ transplant candidacy: A consensus expert opinion statement. *Am J Transplant.* 2021;21(2):460-474.

2. Salcedo M, Vinaixa C, Javle M, Trapero-Marugan M, Bustamante J, Line PD. Evaluation and Management of Liver Transplant Candidates With Prior Nonhepatic Cancer: Guidelines From the ILTS/SETH Consensus Conference. *Transplantation.* 2022;106(1):e3-e11.

3. Banff schema for grading liver allograft rejection: an international consensus document. *Hepatology.* 1997;25(3):658-663.

4. Londono MC, Souza LN, Lozano JJ, et al. Molecular profiling of subclinical inflammatory lesions in long-term surviving adult liver transplant recipients. *J Hepatol.* 2018;69(3):626-634.

5. Wood SN. *Generalized Additive Models. An Introduction with R.* Boca Raton: Chapman and Hall/CRC; 2017.

6. Tibshirani R. Regression Shrinkage and Selection via the Lasso. *Journal of the Royal Statistical Society: Series B.* 1996;58:267-288.

7. Hosmer Jr DW, Lemeshow S, Sturdivant RX. *Applied logistic regression.* 3rd Edition ed. Hoboken, NJ: John Wiley & Sons; 2013.

8. Little RJA, Rubin DB. *Sttistical analysis with missing data.* New York: John Wiley & Sons; 2002.

9. Marshall A, Altman DG, Royston P, Holder RL. Comparison of techniques for handling missing covariate data within prognostic modelling studies: a simulation study. *BMC Med Res Methodol.* 2010;10:7.
