# Supplementary material for: Longitudinal biomarker progression and validation for predicting operational tolerance in a prospective multicenter liver transplantation immunosuppression withdrawal trial
Source: PLoS One. 2025 Dec 8;20(12):e0326442. doi: 10.1371/journal.pone.0326442 (PMC12685220; doi:10.1371/journal.pone.0326442)
Supplement: S2 File — (DOCX) [file pone.0326442.s002.docx]

**Figure S1. - Biomarkers detected in whole blood that show significant differences between TOL and non-TOL groups at each time point during the IS withdrawal protocol.** Only five variables presented a *p*-value below 0.05, including the FOXP3-TSDR methylation ratio (Met_Ratio) at baseline, *SENP6* and *FOXP3* gene expression after 2 months of IS weaning, and *miR95* and *miR31* 12 months after complete IS weaning or a rejection episode. Variables that were more expressed in the non-TOL group are represented in red, while *FOXP3* gene, which was more expressed in TOL patients, is represented in green.

**Figure S2. – Evolution of Tacrolimus and ALT levels during the IS withdrawal protocol.** The blood concentration of Tacrolimus (ng/mL) (a) and the liver enzyme alanine transaminase (ALT – U/L) (b) are shown for both TOL (blue) and non-TOL (red) groups throughout the IS weaning process. Dotted vertical lines indicate the average time elapsed within each study interval. 95% CI represented as shaded bands.

**Figure S3.-** **Probability of belonging to the non-TOL group at different study times.** Linear mixed model with flexible effects were applied for longitudinal analysis of study groups. Among the variables analyzed in whole blood, only *FOXP3*-TSDR methylation rate showed significant differences at the basal point (a), while *FOXP3* expression exhibited an opposite trend (b). The 95% confidence interval is presented as a shaded band, and the time-points analyzed in whole blood are referred to as TO to T5, corresponding to Basal, 2M, 6M, R/TOL, 6M-post R/TOL, and 12M-post R/TOL, respectively.

**Supplementary Figure 4**


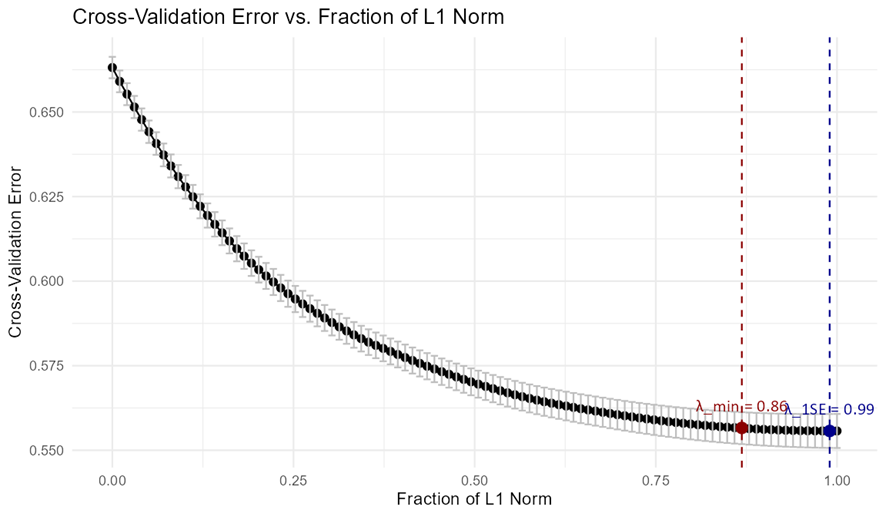


**S4 Fig. Cross-validated error (mean ± 1 standard error) across the sequence of lambda values in the LASSO regression path.** The vertical dashed line on the left indicates the lambda value (λmin) that minimizes the cross-validated error, while the line on the right marks the more parsimonious lambda value (λ1SE) within one standard error of the minimum. The λ1SE model reduces model complexity while maintaining predictive performance close to that of the optimal model.
