# Supplementary material for: Longitudinal biomarker progression and validation for predicting operational tolerance in a prospective multicenter liver transplantation immunosuppression withdrawal trial
Source: PLoS One. 2025 Dec 8;20(12):e0326442. doi: 10.1371/journal.pone.0326442 (PMC12685220; doi:10.1371/journal.pone.0326442)
Supplement: S1 Table — (DOCX) [file pone.0326442.s003.docx]

**Supplementary Table 1.** Biomarkers analyzed.

| **Liver Tissue** | **Measuring units** |
| --- | --- |
| *FOXP3* TSDR-MR | % |
| CD4+FOXP3+ cells | Number/HPF* |
| *HAMP* | Relative expression |
| *SOCS1* | Relative expression |
| *TFRC* | Relative expression |
| *GBP2* | Relative expression |
| *GPNMB* | Relative expression |
| *HLA-DMA* | Relative expression |
| *HMMR* | Relative expression |
| *MMP7* | Relative expression |
| *MMP9* | Relative expression |
| *PLA2G7* | Relative expression |
| **Whole blood** | **Measuring units** |
| *FOXP3* TSDR-MR | % |
| *FEM1C* | Relative expression |
| *FOXP3* | Relative expression |
| *SENP6* | Relative expression |
| *IKZF2* | Relative expression |
| *miR31* | Relative expression |
| *miR95* | Relative expression |

*CD4+FOXP3+ cells quantification was represented as the mean number of double positive cells per high-power field (HPF), averaged from 10 randomly selected high-power fields per image. FOXP3, forkhead box P3; TSDR-MR, regulatory T cell specific demethylated region methylation ratio; HAMP, hepcidine antimicrobial peptide; SOCS1, suppressor of cytokine signaling 1; TFRC, transferrin receptor; GBP2, guanylate binding protein 2; GPNMB, glycoprotein Nmb; HLA-DMA, major histocompatibility complex, Class II, DM alpha; HMMR, hyaluronan mediated motility receptor; MMP, matrix metallopeptidase; PLA2G7, phospholipase A2 group VII; FEM1C, fem-1 homolog C; SENP6, SUMO-specific peptidase 6; IKZF2, IKAROS family zinc finger 2; miR, miRNA.
