# Supplementary material for: Longitudinal biomarker progression and validation for predicting operational tolerance in a prospective multicenter liver transplantation immunosuppression withdrawal trial
Source: PLoS One. 2025 Dec 8;20(12):e0326442. doi: 10.1371/journal.pone.0326442 (PMC12685220; doi:10.1371/journal.pone.0326442)
Supplement: S2 Table — (DOCX) [file pone.0326442.s004.docx]

**Supplementary Table 2.** Patients included per center.

|  | **H1** | **H2** | **H3** | **H4** | **H5** | **H6** | **H7** |
| --- | --- | --- | --- | --- | --- | --- | --- |
| Total enrolled patients | 18 | 48 | 9 | 7 | 4 | 3 | 2 |
| Screen failure | 1 | 11 | 2 | 0 | 1 | 1 | 0 |
| G2 (Control group) | 5 | 12 | 2 | 3 | 1 | 0 | 0 |
| G1 (Study group) | 12 | 25 | 5 | 4 | 2 | 2 | 2 |
| Withdrawn | 3 | 1 | 1 | 1 | 1 | 0 | 0 |
| Finished protocol (Tol; Non-Tol) | 9 (2; 7) | 24 (11; 13) | 4 (3; 1) | 3 (0; 3) | 1 (1; 0) | 2 (0; 2) | 2 (0; 2) |
| % Tol | 22 | 46 | 75 | 0 | 100 | 0 | 0 |
| % Non-Tol | 78 | 34 | 25 | 100 | 0 | 100 | 100 |
| ^†^Basal Tacrolimus concentration (ng/ml)** | 4.77 ± 1.08 | 2.77 ± 1.49 | 2.43 ± 1.12 | 5.7 ± 0.75 | 1.6 | 3.75 ± 2.47 | 2.35 ± 0.21 |

^†^Only for the 45 patients who finished the trial. **P < 0.002 as calculated by a one-way Anova test.
