# Supplementary material for: Longitudinal biomarker progression and validation for predicting operational tolerance in a prospective multicenter liver transplantation immunosuppression withdrawal trial
Source: PLoS One. 2025 Dec 8;20(12):e0326442. doi: 10.1371/journal.pone.0326442 (PMC12685220; doi:10.1371/journal.pone.0326442)
Supplement: S3 Table — (DOCX) [file pone.0326442.s005.docx]

**Supplementary Table 3.** Comparison of demographic and clinical characteristics between control and study groups.

|  | **G1 (n = 52)** | **G2 (n = 23)** | ***P*** |
| --- | --- | --- | --- |
| Age [median (range)] | [63 (42-75)] | [62 (28-75)] | 0.405^a^ |
| Age at transplantation [median (range)] | [54 (22-69)] | [52 (19-71)] | 0.511^a^ |
| Months from transplant to weaning start [median (range)] | [91 (37-260)] | [99 (40-221)] | 0.830^a^ |
| Gender (n; %) |  |  | 0.657^b^ |
| Male | (45; 87) | (19; 83) |  |
| Female | (7; 13) | (4; 17) |  |
| Co-morbid medical problems (n;%) |  |  |  |
| Diabetes | (28; 54) | (5; 22) | **0.010** ^b^ |
| Hypertension | (41; 79) | (14; 61) | 0.105^b^ |
| Cardiovascular pathology | (11; 21) | (2; 9) | 0.189^b^ |
| Renal dysfunction | (17; 33) | (5; 22) | 0.313^b^ |
| Diseases (n; %) |  |  |  |
| Alcoholic cirrhosis | (18; 35) | (5; 22) | 0.265^b^ |
| Alcoholic cirrhosis + HCC | (7; 13) | (4; 17) | 0.667^b^ |
| HCV cirrhosis | (12; 23) | (6; 26) | 0.778^b^ |
| HCV cirrhosis + HCC | (8; 15) | (6; 26) | 0.273^b^ |
| Other | (9; 17) | (2; 9) | 0.331^b^ |
| Basal LFTs [median (range)] |  |  |  |
| ALT (U/L) | [17.5 (5-46] | [17 (9-95)] | 0.673^a^ |
| ALP (U/L) | [68 (35-141)] | [66 (33-96)] | 0.130^a^ |
| Total Bilirrubin (mg/dL) | [0.5 (0.19-2.66)] | [0.62 (0.19-1.27)] | 0.979^a^ |
| INR | [1.01 (0.9-1.3)] | [1 (0.9-1.31 9] | 0.974^a^ |
| Main IS drug (n; %) |  |  |  |
| Cyclosporine A | 3 (6) | 2 (9) | 0.639^b^ |
| Tacrolimus | 16 (31) | 10 (43) | 0.286^b^ |
| Tacrolimus + MMF | 27 (52) | 9 (39) | 0.128^b^ |
| MMF | 6 (11) | 2 (9) | 0.713^b^ |
| Basal Tacrolimus concentration (ng/ml) [median (range)] |  |  |  |
| Tacrolimus | [3.9 (1-7.6)] | [5.3 (2.1-8.1)] | 0.051^a^ |
| Tacrolimus + MMF | [2.7 (0.6-8.8)] | [2.1 (0.7-4.4)] | 0.189^a^ |

^a^T-test. ^b^Chi-square test. HCC: Hepatocellular carcinoma; HCV: Hepatitis C virus; LFTs: liver function tests; ALT: Alanine aminotransferase; ALP: Alkaline phosphatase; INR: International normalized ratio; MMF: Mycophenolate mofetil..
