# Supplementary material for: Longitudinal biomarker progression and validation for predicting operational tolerance in a prospective multicenter liver transplantation immunosuppression withdrawal trial
Source: PLoS One. 2025 Dec 8;20(12):e0326442. doi: 10.1371/journal.pone.0326442 (PMC12685220; doi:10.1371/journal.pone.0326442)
Supplement: S4 Table — (DOCX) [file pone.0326442.s006.docx]

**Supplementary Table 4.-** Statistical analysis of different variables evaluated in whole blood between TOL and non-TOL groups at different time points.

**Basal**

| **Variable** | **Non-Tol (N=28)** | **Tol (N=17)** | **Total (N=45)** | **Test** | **Statistic** | **p-value** |
| --- | --- | --- | --- | --- | --- | --- |
| TSDR-Foxp3 methylation (%) |  |  |  | Mann-Whitney | W = 293.500 | **0,038** |
| - N | 25 | 17 | 42 |  |  |  |
| - Mean (SD) | 90.89 (1.03) | 89.85 (1.91) | 90.47 (1.52) |  |  |  |
| - Median (Q1, Q3) | 91.07 (90.52, 91.36) | 90.30 (89.74, 91.15) | 90.87 (90.18, 91.33) |  |  |  |
| - Range | 88.27 - 92.44 | 84.56 - 91.93 | 84.56 - 92.44 |  |  |  |
| fem1c (Relative units) |  |  |  | Mann-Whitney | W = 254.000 | 0,242 |
| - N | 26 | 16 | 42 |  |  |  |
| - Mean (SD) | 2.67 (2.69) | 2.00 (2.72) | 2.41 (2.69) |  |  |  |
| - Median (Q1, Q3) | 1.77 (1.09, 3.30) | 1.42 (0.71, 1.85) | 1.58 (1.07, 2.50) |  |  |  |
| - Range | 0.22 - 10.47 | 0.32 - 11.77 | 0.22 - 11.77 |  |  |  |
| senp6 (Relative units) |  |  |  | Mann-Whitney | W = 221.000 | 1 |
| - N | 26 | 17 | 43 |  |  |  |
| - Mean (SD) | 2.49 (1.48) | 2.22 (0.80) | 2.38 (1.25) |  |  |  |
| - Median (Q1, Q3) | 1.98 (1.47, 3.23) | 2.07 (1.75, 2.87) | 2.05 (1.48, 3.05) |  |  |  |
| - Range | 0.77 - 6.73 | 1.09 - 3.50 | 0.77 - 6.73 |  |  |  |
| foxp3 (Relative units) |  |  |  | t-test | t (39) = -1.381 | 0,175 |
| - N | 24 | 17 | 41 |  |  |  |
| - Mean (SD) | 0.03 (0.02) | 0.04 (0.02) | 0.04 (0.02) |  |  |  |
| - Median (Q1, Q3) | 0.03 (0.02, 0.05) | 0.04 (0.03, 0.05) | 0.04 (0.03, 0.05) |  |  |  |
| - Range | 0.01 - 0.07 | 0.01 - 0.10 | 0.01 - 0.10 |  |  |  |
| ikf2 (Relative units) |  |  |  | Mann-Whitney | W = 129.000 | 0,071 |
| - N | 23 | 17 | 40 |  |  |  |
| - Mean (SD) | 0.10 (0.08) | 0.13 (0.08) | 0.11 (0.08) |  |  |  |
| - Median (Q1, Q3) | 0.07 (0.05, 0.11) | 0.10 (0.09, 0.14) | 0.09 (0.06, 0.13) |  |  |  |
| - Range | 0.02 - 0.34 | 0.04 - 0.34 | 0.02 - 0.34 |  |  |  |
| mi_r31 (Relative units) |  |  |  | Mann-Whitney | W = 289.000 | 0,232 |
| - N | 28 | 17 | 45 |  |  |  |
| - Mean (SD) | 3.77 (10.62) | 0.25 (0.31) | 2.44 (8.50) |  |  |  |
| - Median (Q1, Q3) | 0.20 (0.04, 1.04) | 0.09 (0.03, 0.44) | 0.15 (0.03, 0.49) |  |  |  |
| - Range | 0.00 - 47.21 | 0.00 - 0.96 | 0.00 - 47.21 |  |  |  |
| mi_r95 (Relative units) |  |  |  | Mann-Whitney | W = 289.000 | 0,152 |
| - N | 27 | 17 | 44 |  |  |  |
| - Mean (SD) | 1.28 (3.88) | 0.13 (0.26) | 0.83 (3.07) |  |  |  |
| - Median (Q1, Q3) | 0.06 (0.01, 0.20) | 0.01 (0.00, 0.06) | 0.04 (0.00, 0.10) |  |  |  |
| - Range | 0.00 - 17.11 | 0.00 - 0.80 | 0.00 - 17.11 |  |  |  |

**2M**

| **Variable** | **Non-Tol (N=28)** | **Tol (N=17)** | **Total (N=45)** | **Test** | **Statistic** | **p-value** |
| --- | --- | --- | --- | --- | --- | --- |
| TSDR-Foxp3 methylation (%) |  |  |  | Mann-Whitney | W = 241.000 | 0,273 |
| - N | 25 | 16 | 41 |  |  |  |
| - Mean (SD) | 90.94 (1.29) | 90.44 (1.46) | 90.75 (1.36) |  |  |  |
| - Median (Q1, Q3) | 91.29 (90.75, 91.71) | 90.82 (89.35, 91.65) | 91.14 (90.46, 91.65) |  |  |  |
| - Range | 87.26 - 92.65 | 87.48 - 92.13 | 87.26 - 92.65 |  |  |  |
| fem1c (Relative units) |  |  |  | Mann-Whitney | W = 208.000 | 0,843 |
| - N | 25 | 16 | 41 |  |  |  |
| - Mean (SD) | 1.46 (1.09) | 1.46 (1.15) | 1.46 (1.10) |  |  |  |
| - Median (Q1, Q3) | 1.09 (0.65, 2.11) | 1.15 (0.63, 1.89) | 1.09 (0.64, 2.11) |  |  |  |
| - Range | 0.28 - 4.42 | 0.18 - 3.98 | 0.18 - 4.42 |  |  |  |
| senp6 (Relative units) |  |  |  | Mann-Whitney | W = 274.000 | 0,049 |
| - N | 25 | 16 | 41 |  |  |  |
| - Mean (SD) | 2.51 (2.01) | 1.71 (0.76) | 2.20 (1.67) |  |  |  |
| - Median (Q1, Q3) | 2.06 (1.59, 2.42) | 1.67 (1.26, 1.96) | 1.93 (1.38, 2.18) |  |  |  |
| - Range | 0.97 - 10.84 | 0.52 - 3.39 | 0.52 - 10.84 |  |  |  |
| foxp3 (Relative units) |  |  |  | Mann-Whitney | W = 119.000 | 0,03 |
| - N | 25 | 16 | 41 |  |  |  |
| - Mean (SD) | 0.03 (0.02) | 0.04 (0.02) | 0.04 (0.02) |  |  |  |
| - Median (Q1, Q3) | 0.03 (0.01, 0.04) | 0.05 (0.03, 0.06) | 0.03 (0.02, 0.05) |  |  |  |
| - Range | 0.00 - 0.10 | 0.00 - 0.09 | 0.00 - 0.10 |  |  |  |
| ikf2 (Relative units) |  |  |  | Mann-Whitney | W = 205.000 | 0,639 |
| - N | 25 | 15 | 40 |  |  |  |
| - Mean (SD) | 0.09 (0.07) | 0.08 (0.03) | 0.09 (0.06) |  |  |  |
| - Median (Q1, Q3) | 0.08 (0.04, 0.12) | 0.07 (0.06, 0.10) | 0.07 (0.05, 0.11) |  |  |  |
| - Range | 0.00 - 0.28 | 0.02 - 0.14 | 0.00 - 0.28 |  |  |  |
| mi_r31 (Relative units) |  |  |  | Mann-Whitney | W = 215.000 | 0,525 |
| - N | 24 | 16 | 40 |  |  |  |
| - Mean (SD) | 1.59 (3.09) | 6.80 (19.44) | 3.67 (12.56) |  |  |  |
| - Median (Q1, Q3) | 0.10 (0.03, 1.37) | 0.08 (0.03, 0.29) | 0.09 (0.03, 0.97) |  |  |  |
| - Range | 0.01 - 12.30 | 0.01 - 72.48 | 0.01 - 72.48 |  |  |  |
| mi_r95 (Relative units) |  |  |  | Mann-Whitney | W = 258.000 | 0,121 |
| - N | 25 | 16 | 41 |  |  |  |
| - Mean (SD) | 2.15 (8.35) | 5.67 (21.59) | 3.52 (14.82) |  |  |  |
| - Median (Q1, Q3) | 0.03 (0.00, 0.56) | 0.00 (0.00, 0.04) | 0.02 (0.00, 0.43) |  |  |  |
| - Range | 0.00 - 41.92 | 0.00 - 86.57 | 0.00 - 86.57 |  |  |  |

**6M**

| **Variable** | **Non-Tol (N=28)** | **Tol (N=17)** | **Total (N=45)** | **Test** | **Statistic** | **p-value** |
| --- | --- | --- | --- | --- | --- | --- |
| TSDR-Foxp3 methylation (%) |  |  |  | Mann-Whitney | W = 153.000 | 0,532 |
| - N | 18 | 15 | 33 |  |  |  |
| - Mean (SD) | 90.93 (1.20) | 90.65 (1.36) | 90.80 (1.26) |  |  |  |
| - Median (Q1, Q3) | 91.18 (90.65, 91.66) | 91.05 (90.56, 91.44) | 91.05 (90.63, 91.52) |  |  |  |
| - Range | 88.33 - 92.40 | 87.59 - 92.09 | 87.59 - 92.40 |  |  |  |
| fem1c (Relative units) |  |  |  | Mann-Whitney | W = 167.000 | 0,75 |
| - N | 21 | 17 | 38 |  |  |  |
| - Mean (SD) | 1.18 (0.90) | 1.53 (1.96) | 1.34 (1.46) |  |  |  |
| - Median (Q1, Q3) | 1.00 (0.50, 1.39) | 1.15 (0.54, 1.46) | 1.03 (0.51, 1.42) |  |  |  |
| - Range | 0.41 - 4.06 | 0.30 - 8.71 | 0.30 - 8.71 |  |  |  |
| senp6 (Relative units) |  |  |  | Mann-Whitney | W = 153.000 | 0,467 |
| - N | 21 | 17 | 38 |  |  |  |
| - Mean (SD) | 2.25 (1.41) | 3.12 (2.39) | 2.64 (1.94) |  |  |  |
| - Median (Q1, Q3) | 2.02 (1.41, 2.55) | 2.07 (1.38, 4.78) | 2.03 (1.39, 2.95) |  |  |  |
| - Range | 0.36 - 6.40 | 0.88 - 8.57 | 0.36 - 8.57 |  |  |  |
| foxp3 (Relative units) |  |  |  | Mann-Whitney | W = 162.000 | 0,642 |
| - N | 21 | 17 | 38 |  |  |  |
| - Mean (SD) | 0.04 (0.04) | 0.04 (0.03) | 0.04 (0.03) |  |  |  |
| - Median (Q1, Q3) | 0.03 (0.01, 0.06) | 0.03 (0.02, 0.05) | 0.03 (0.01, 0.05) |  |  |  |
| - Range | 0.00 - 0.13 | 0.00 - 0.11 | 0.00 - 0.13 |  |  |  |
| ikf2 (Relative units) |  |  |  | Mann-Whitney | W = 107.000 | 0,087 |
| - N | 19 | 17 | 36 |  |  |  |
| - Mean (SD) | 0.08 (0.06) | 0.15 (0.13) | 0.11 (0.10) |  |  |  |
| - Median (Q1, Q3) | 0.05 (0.03, 0.13) | 0.08 (0.05, 0.25) | 0.08 (0.03, 0.16) |  |  |  |
| - Range | 0.02 - 0.17 | 0.02 - 0.49 | 0.02 - 0.49 |  |  |  |
| mi_r31 (Relative units) |  |  |  | Mann-Whitney | W = 149.000 | 0,885 |
| - N | 22 | 14 | 36 |  |  |  |
| - Mean (SD) | 0.24 (0.56) | 10.99 (39.97) | 4.42 (24.94) |  |  |  |
| - Median (Q1, Q3) | 0.06 (0.02, 0.14) | 0.06 (0.02, 0.24) | 0.06 (0.02, 0.24) |  |  |  |
| - Range | 0.01 - 2.62 | 0.01 - 149.83 | 0.01 - 149.83 |  |  |  |
| mi_r95 (Relative units) |  |  |  | Mann-Whitney | W = 139.000 | 0,906 |
| - N | 22 | 13 | 35 |  |  |  |
| - Mean (SD) | 0.09 (0.25) | 0.10 (0.30) | 0.10 (0.27) |  |  |  |
| - Median (Q1, Q3) | 0.01 (0.00, 0.04) | 0.01 (0.00, 0.04) | 0.01 (0.00, 0.04) |  |  |  |
| - Range | 0.00 - 1.05 | 0.00 - 1.09 | 0.00 - 1.09 |  |  |  |

**R/Tol**

| **Variable** | **Tol** | **non-Tol** |
| --- | --- | --- |
| TSDR-Foxp3 methylation (%) |  |  |
| - (Missing) | 1 | 12 |
| - Mean (SD) | 90.09 (1.27) | 89.95 (1.32) |
| - Min - Max | 87.82 - 91.88 | 87.67 - 92.22 |
| fem1c (Relative units) |  |  |
| - (Missing) | 2 | 11 |
| - Mean (SD) | 1.78 (2.61) | 1.13 (1.09) |
| - Min - Max | 0.34 - 10.18 | 0.41 - 4.98 |
| senp6 (Relative units) |  |  |
| - (Missing) | 2 | 11 |
| - Mean (SD) | 2.60 (2.89) | 2.21 (0.90) |
| - Min - Max | 0.85 - 12.63 | 1.07 - 4.22 |
| foxp3 (Relative units) |  |  |
| - (Missing) | 1 | 11 |
| - Mean (SD) | 0.02 (0.01) | 0.04 (0.02) |
| - Min - Max | 0.00 - 0.05 | 0.00 - 0.08 |
| ikf2 (Relative units) |  |  |
| - (Missing) | 2 | 11 |
| - Mean (SD) | 0.06 (0.07) | 0.07 (0.04) |
| - Min - Max | 0.00 - 0.28 | 0.02 - 0.19 |
| mi_r31 (Relative units) |  |  |
| - (Missing) | 1 | 10 |
| - Mean (SD) | 0.14 (0.08) | 0.51 (0.99) |
| - Min - Max | 0.01 - 0.25 | 0.02 - 3.38 |
| mi_r95 (Relative units) |  |  |
| - (Missing) | 1 | 10 |
| - Mean (SD) | 0.01 (0.01) | 0.46 (1.42) |
| - Min - Max | 0.00 - 0.04 | 0.00 - 5.99 |

**6M-post**

| **Variable** | **Non-Tol (N=28)** | **Tol (N=17)** | **Total (N=45)** | **Test** | **Statistic** | **p-value** |
| --- | --- | --- | --- | --- | --- | --- |
| TSDR-Foxp3 methylation (%) |  |  |  | t-test | t (24) = -1.173 | 0,252 |
| - N | 14 | 12 | 26 |  |  |  |
| - Mean (SD) | 90.58 (0.79) | 91.08 (1.37) | 90.81 (1.10) |  |  |  |
| - Median (Q1, Q3) | 90.68 (90.12, 91.11) | 91.25 (90.36, 91.66) | 90.85 (90.29, 91.33) |  |  |  |
| - Range | 89.14 - 91.84 | 89.11 - 94.06 | 89.11 - 94.06 |  |  |  |
| fem1c (Relative units) |  |  |  | Mann-Whitney | W = 100.000 | 0,577 |
| - N | 16 | 11 | 27 |  |  |  |
| - Mean (SD) | 1.45 (1.60) | 1.16 (1.12) | 1.33 (1.41) |  |  |  |
| - Median (Q1, Q3) | 0.89 (0.54, 1.54) | 0.69 (0.50, 1.42) | 0.85 (0.52, 1.47) |  |  |  |
| - Range | 0.29 - 6.60 | 0.35 - 4.24 | 0.29 - 6.60 |  |  |  |
| senp6 (Relative units) |  |  |  | Mann-Whitney | W = 108.000 | 0,342 |
| - N | 16 | 11 | 27 |  |  |  |
| - Mean (SD) | 3.07 (2.57) | 1.94 (0.96) | 2.61 (2.12) |  |  |  |
| - Median (Q1, Q3) | 2.05 (1.75, 3.27) | 1.91 (1.38, 2.39) | 1.93 (1.54, 2.89) |  |  |  |
| - Range | 1.33 - 10.99 | 0.10 - 3.65 | 0.10 - 10.99 |  |  |  |
| foxp3 (Relative units) |  |  |  | Mann-Whitney | W = 58.000 | 0,148 |
| - N | 16 | 11 | 27 |  |  |  |
| - Mean (SD) | 0.03 (0.05) | 0.02 (0.02) | 0.03 (0.04) |  |  |  |
| - Median (Q1, Q3) | 0.01 (0.01, 0.03) | 0.01 (0.01, 0.04) | 0.01 (0.01, 0.04) |  |  |  |
| - Range | 0.00 - 0.19 | 0.01 - 0.06 | 0.00 - 0.19 |  |  |  |
| ikf2 (Relative units) |  |  |  | Mann-Whitney | W = 117.000 | 0,162 |
| - N | 16 | 11 | 27 |  |  |  |
| - Mean (SD) | 0.07 (0.07) | 0.04 (0.03) | 0.06 (0.06) |  |  |  |
| - Median (Q1, Q3) | 0.05 (0.03, 0.06) | 0.03 (0.02, 0.04) | 0.04 (0.02, 0.06) |  |  |  |
| - Range | 0.01 - 0.23 | 0.02 - 0.11 | 0.01 - 0.23 |  |  |  |
| mi_r31 (Relative units) |  |  |  | Mann-Whitney | W = 101.000 | 0,837 |
| - N | 16 | 12 | 28 |  |  |  |
| - Mean (SD) | 0.29 (0.38) | 2.85 (7.51) | 1.39 (4.97) |  |  |  |
| - Median (Q1, Q3) | 0.15 (0.10, 0.23) | 0.13 (0.05, 0.55) | 0.15 (0.09, 0.27) |  |  |  |
| - Range | 0.04 - 1.43 | 0.01 - 26.14 | 0.01 - 26.14 |  |  |  |
| mi_r95 (Relative units) |  |  |  | Mann-Whitney | W = 91.000 | 0,904 |
| - N | 16 | 11 | 27 |  |  |  |
| - Mean (SD) | 0.12 (0.32) | 0.42 (1.15) | 0.24 (0.77) |  |  |  |
| - Median (Q1, Q3) | 0.02 (0.01, 0.05) | 0.02 (0.01, 0.10) | 0.02 (0.01, 0.07) |  |  |  |
| - Range | 0.00 - 1.30 | 0.00 - 3.87 | 0.00 - 3.87 |  |  |  |

**12M-post**

| **Variable** | **Non-Tol (N=28)** | **Tol (N=17)** | **Total (N=45)** | **Test** | **Statistic** | **p-value** |
| --- | --- | --- | --- | --- | --- | --- |
| TSDR-Foxp3 methylation (%) |  |  |  | t-test | t (30) = 1.121 | 0,271 |
| - N | 16 | 16 | 32 |  |  |  |
| - Mean (SD) | 91.04 (2.41) | 90.02 (2.72) | 90.53 (2.58) |  |  |  |
| - Median (Q1, Q3) | 91.07 (89.84, 92.10) | 90.48 (88.74, 91.18) | 90.74 (89.50, 91.88) |  |  |  |
| - Range | 85.71 - 95.89 | 84.90 - 95.96 | 84.90 - 95.96 |  |  |  |
| fem1c (Relative units) |  |  |  | Mann-Whitney | W = 150.000 | 0,126 |
| - N | 15 | 15 | 30 |  |  |  |
| - Mean (SD) | 2.46 (2.41) | 1.69 (2.40) | 2.08 (2.39) |  |  |  |
| - Median (Q1, Q3) | 1.54 (0.72, 3.16) | 0.66 (0.42, 1.67) | 0.77 (0.57, 2.78) |  |  |  |
| - Range | 0.36 - 8.10 | 0.24 - 9.02 | 0.24 - 9.02 |  |  |  |
| senp6 (Relative units) |  |  |  | Mann-Whitney | W = 141.000 | 0,423 |
| - N | 15 | 16 | 31 |  |  |  |
| - Mean (SD) | 3.18 (1.86) | 2.69 (1.84) | 2.93 (1.84) |  |  |  |
| - Median (Q1, Q3) | 2.74 (1.85, 4.45) | 2.48 (0.98, 3.81) | 2.70 (1.57, 4.43) |  |  |  |
| - Range | 1.35 - 8.31 | 0.37 - 6.67 | 0.37 - 8.31 |  |  |  |
| foxp3 (Relative units) |  |  |  | Mann-Whitney | W = 113.000 | 0,8 |
| - N | 15 | 16 | 31 |  |  |  |
| - Mean (SD) | 0.03 (0.02) | 0.03 (0.03) | 0.03 (0.03) |  |  |  |
| - Median (Q1, Q3) | 0.03 (0.01, 0.03) | 0.02 (0.01, 0.04) | 0.02 (0.01, 0.03) |  |  |  |
| - Range | 0.00 - 0.08 | 0.00 - 0.12 | 0.00 - 0.12 |  |  |  |
| ikf2 (Relative units) |  |  |  | Mann-Whitney | W = 167.000 | 0,149 |
| - N | 16 | 16 | 32 |  |  |  |
| - Mean (SD) | 0.07 (0.04) | 0.07 (0.09) | 0.07 (0.07) |  |  |  |
| - Median (Q1, Q3) | 0.06 (0.05, 0.09) | 0.04 (0.03, 0.06) | 0.05 (0.04, 0.08) |  |  |  |
| - Range | 0.01 - 0.15 | 0.02 - 0.39 | 0.01 - 0.39 |  |  |  |
| mi_r31 (Relative units) |  |  |  | Mann-Whitney | W = 190.000 | **0,019** |
| - N | 16 | 16 | 32 |  |  |  |
| - Mean (SD) | 3.89 (7.31) | 0.91 (2.38) | 2.40 (5.56) |  |  |  |
| - Median (Q1, Q3) | 0.35 (0.20, 3.51) | 0.10 (0.05, 0.26) | 0.25 (0.07, 0.40) |  |  |  |
| - Range | 0.02 - 26.05 | 0.00 - 9.25 | 0.00 - 26.05 |  |  |  |
| mi_r95 (Relative units) |  |  |  | Mann-Whitney | W = 186.000 | **0,029** |
| - N | 16 | 16 | 32 |  |  |  |
| - Mean (SD) | 2.30 (5.39) | 0.25 (0.80) | 1.28 (3.93) |  |  |  |
| - Median (Q1, Q3) | 0.04 (0.02, 0.73) | 0.01 (0.01, 0.03) | 0.02 (0.01, 0.13) |  |  |  |
| - Range | 0.00 - 16.55 | 0.00 - 3.24 | 0.00 - 16.55 |  |  |  |

**Odds ratio**

| **Variables** | **REJECTION N=16** | **TOLERANCE N=16** | **OR** | **p.ratio** |
| --- | --- | --- | --- | --- |
| TSDR-Foxp3 methylation (%) | 91.0 (2.41) | 90.0 (2.72) | 0.85 [0.63;1.14] | 0,267 |
| fem1c (Relative units) | 1.54 [0.72;3.16] | 0.66 [0.42;1.67] | 0.87 [0.63;1.20] | 0,381 |
| senp6 (Relative units) | 2.74 [1.85;4.45] | 2.48 [0.98;3.81] | 0.86 [0.57;1.28] | 0,448 |
| foxp3 (Relative units) | 0.03 [0.01;0.03] | 0.02 [0.01;0.04] | 8.339000e+03 [0.000000e+00;6.695921e+15] | 0,519 |
| ikf2 (Relative units) | 0.06 [0.05;0.09] | 0.04 [0.03;0.06] | 0.69 [0.00;29334] | 0,945 |
| mi_r31 (Relative units) | 0.35 [0.20;3.51] | 0.10 [0.05;0.26] | 0.86 [0.69;1.08] | 0,193 |
| mi_r95 (Relative units) | 0.04 [0.02;0.73] | 0.01 [0.01;0.03] | 0.76 [0.42;1.37] | 0,359 |
