# Supplementary material for: Longitudinal biomarker progression and validation for predicting operational tolerance in a prospective multicenter liver transplantation immunosuppression withdrawal trial
Source: PLoS One. 2025 Dec 8;20(12):e0326442. doi: 10.1371/journal.pone.0326442 (PMC12685220; doi:10.1371/journal.pone.0326442)
Supplement: S5 Table — (DOCX) [file pone.0326442.s007.docx]

**Supplementary Table 5.-** Statistical analysis of different variables evaluated in liver tissue between TOL and non-TOL groups at different time points.

**Basal**

| **Variable** | **Non-Tol (N=28)** | **Tol (N=17)** | **Total (N=45)** | **Test** | **Statistic** | **p-value** |
| --- | --- | --- | --- | --- | --- | --- |
| TSDR-Foxp3 methylation (%) |  |  |  | Mann-Whitney | W = 209.500 | 0,939 |
| - N | 25 | 17 | 42 |  |  |  |
| - Mean (SD) | 88.71 (3.00) | 89.07 (1.90) | 88.86 (2.59) |  |  |  |
| - Median (Q1, Q3) | 89.58 (87.26, 90.81) | 89.26 (88.63, 90.34) | 89.37 (87.59, 90.69) |  |  |  |
| - Range | 80.76 - 92.86 | 84.49 - 91.47 | 80.76 - 92.86 |  |  |  |
| hamp_1 (Relative units) |  |  |  | Mann-Whitney | W = 169.000 | 0,203 |
| - N | 26 | 17 | 43 |  |  |  |
| - Mean (SD) | 5.14 (9.25) | 4.78 (4.50) | 4.99 (7.66) |  |  |  |
| - Median (Q1, Q3) | 2.16 (0.80, 3.78) | 3.32 (1.89, 6.57) | 2.39 (0.98, 4.77) |  |  |  |
| - Range | 0.09 - 39.28 | 0.05 - 15.97 | 0.05 - 39.28 |  |  |  |
| socs_1 (Relative units) |  |  |  | Mann-Whitney | W = 247.000 | 0,531 |
| - N | 26 | 17 | 43 |  |  |  |
| - Mean (SD) | 0.89 (2.93) | 0.36 (0.59) | 0.68 (2.30) |  |  |  |
| - Median (Q1, Q3) | 0.04 (0.01, 0.39) | 0.02 (0.01, 0.47) | 0.03 (0.01, 0.45) |  |  |  |
| - Range | 0.00 - 14.98 | 0.00 - 1.77 | 0.00 - 14.98 |  |  |  |
| tfrc_1 (Relative units) |  |  |  | Mann-Whitney | W = 224.000 | 0,162 |
| - N | 22 | 16 | 38 |  |  |  |
| - Mean (SD) | 0.09 (0.11) | 0.05 (0.05) | 0.08 (0.09) |  |  |  |
| - Median (Q1, Q3) | 0.05 (0.03, 0.12) | 0.03 (0.02, 0.05) | 0.03 (0.02, 0.11) |  |  |  |
| - Range | 0.01 - 0.48 | 0.01 - 0.20 | 0.01 - 0.48 |  |  |  |
| CD4+Foxp3+ cells (number/HPF) |  |  |  | Mann-Whitney | W = 121.000 | 0,445 |
| - N | 19 | 15 | 34 |  |  |  |
| - Mean (SD) | 4.89 (9.13) | 5.13 (5.83) | 5.00 (7.74) |  |  |  |
| - Median (Q1, Q3) | 2.00 (0.00, 5.50) | 3.00 (0.50, 7.50) | 2.50 (0.00, 6.00) |  |  |  |
| - Range | 0.00 - 38.00 | 0.00 - 17.00 | 0.00 - 38.00 |  |  |  |
| gbp2 (Relative units) |  |  |  | Mann-Whitney | W = 142.000 | 0,582 |
| - N | 20 | 16 | 36 |  |  |  |
| - Mean (SD) | 4.72 (7.32) | 4.61 (5.53) | 4.67 (6.50) |  |  |  |
| - Median (Q1, Q3) | 1.36 (0.56, 3.34) | 2.62 (0.61, 6.01) | 1.71 (0.59, 5.66) |  |  |  |
| - Range | 0.00 - 22.31 | 0.02 - 17.08 | 0.00 - 22.31 |  |  |  |
| gpnmb (Relative units) |  |  |  | Mann-Whitney | W = 139.000 | 0,901 |
| - N | 18 | 15 | 33 |  |  |  |
| - Mean (SD) | 0.72 (1.05) | 0.39 (0.53) | 0.57 (0.86) |  |  |  |
| - Median (Q1, Q3) | 0.16 (0.06, 0.95) | 0.14 (0.07, 0.48) | 0.14 (0.07, 0.70) |  |  |  |
| - Range | 0.00 - 3.29 | 0.03 - 1.68 | 0.00 - 3.29 |  |  |  |
| hla_dma (Relative units) |  |  |  | Mann-Whitney | W = 165.000 | 0,547 |
| - N | 22 | 17 | 39 |  |  |  |
| - Mean (SD) | 3.91 (5.16) | 5.43 (7.91) | 4.57 (6.45) |  |  |  |
| - Median (Q1, Q3) | 2.01 (0.82, 5.64) | 2.57 (0.97, 4.75) | 2.17 (0.92, 5.42) |  |  |  |
| - Range | 0.03 - 22.70 | 0.09 - 32.45 | 0.03 - 32.45 |  |  |  |
| hmmr (Relative units) |  |  |  | Mann-Whitney | W = 163.000 | 0,436 |
| - N | 20 | 14 | 34 |  |  |  |
| - Mean (SD) | 2.19 (3.89) | 1.48 (2.81) | 1.90 (3.46) |  |  |  |
| - Median (Q1, Q3) | 0.97 (0.20, 1.94) | 0.58 (0.19, 1.18) | 0.65 (0.16, 1.91) |  |  |  |
| - Range | 0.05 - 16.89 | 0.03 - 10.82 | 0.03 - 16.89 |  |  |  |
| mmp9 (Relative units) |  |  |  | Mann-Whitney | W = 91.000 | 0,115 |
| - N | 10 | 13 | 23 |  |  |  |
| - Mean (SD) | 0.13 (0.15) | 0.05 (0.06) | 0.08 (0.12) |  |  |  |
| - Median (Q1, Q3) | 0.04 (0.02, 0.25) | 0.02 (0.01, 0.11) | 0.02 (0.01, 0.13) |  |  |  |
| - Range | 0.01 - 0.40 | 0.00 - 0.15 | 0.00 - 0.40 |  |  |  |
| pla2g7 (Relative units) |  |  |  | Mann-Whitney | W = 145.000 | 0,882 |
| - N | 20 | 15 | 35 |  |  |  |
| - Mean (SD) | 1.54 (2.54) | 1.18 (1.66) | 1.38 (2.19) |  |  |  |
| - Median (Q1, Q3) | 0.49 (0.12, 1.45) | 0.43 (0.20, 1.32) | 0.43 (0.14, 1.56) |  |  |  |
| - Range | 0.01 - 9.78 | 0.06 - 6.13 | 0.01 - 9.78 |  |  |  |
| mmp7 (Relative units) |  |  |  | Mann-Whitney | W = 155.000 | 0,803 |
| - N | 21 | 14 | 35 |  |  |  |
| - Mean (SD) | 4.85 (9.07) | 3.81 (6.73) | 4.43 (8.12) |  |  |  |
| - Median (Q1, Q3) | 0.48 (0.15, 4.25) | 0.41 (0.12, 4.63) | 0.48 (0.13, 4.88) |  |  |  |
| - Range | 0.05 - 36.31 | 0.03 - 23.34 | 0.03 - 36.31 |  |  |  |

**Rejection**

| **Variable** | **Non-Tol (N=28)** | **Total (N=28)** |
| --- | --- | --- |
| TSDR-Foxp3 methylation (%) |  |  |
| - (Missing) | 13 | 13 |
| - Mean (SD) | 90.52 (2.82) | 90.52 (2.82) |
| - Min - Max | 85.00 - 95.07 | 85.00 - 95.07 |
| hamp_1 (Relative units) |  |  |
| - (Missing) | 13 | 13 |
| - Mean (SD) | 5.04 (6.16) | 5.04 (6.16) |
| - Min - Max | 0.29 - 18.44 | 0.29 - 18.44 |
| socs_1 (Relative units) |  |  |
| - (Missing) | 14 | 14 |
| - Mean (SD) | 0.58 (1.21) | 0.58 (1.21) |
| - Min - Max | 0.00 - 3.87 | 0.00 - 3.87 |
| tfrc_1 (Relative units) |  |  |
| - (Missing) | 13 | 13 |
| - Mean (SD) | 0.14 (0.18) | 0.14 (0.18) |
| - Min - Max | 0.01 - 0.64 | 0.01 - 0.64 |
| CD4+Foxp3+ cells (number) |  |  |
| - (Missing) | 25 | 25 |
| - Mean (SD) | 33.67 (26.63) | 33.67 (26.63) |
| - Min - Max | 3.00 - 51.00 | 3.00 - 51.00 |
| gbp2 (Relative units) |  |  |
| - (Missing) | 16 | 16 |
| - Mean (SD) | 1.68 (1.70) | 1.68 (1.70) |
| - Min - Max | 0.01 - 4.42 | 0.01 - 4.42 |
| gpnmb (Relative units) |  |  |
| - (Missing) | 18 | 18 |
| - Mean (SD) | 0.32 (0.32) | 0.32 (0.32) |
| - Min - Max | 0.02 - 1.07 | 0.02 - 1.07 |
| hla_dma (Relative units) |  |  |
| - (Missing) | 15 | 15 |
| - Mean (SD) | 6.88 (8.33) | 6.88 (8.33) |
| - Min - Max | 0.50 - 23.57 | 0.50 - 23.57 |
| hmmr (Relative units) |  |  |
| - (Missing) | 15 | 15 |
| - Mean (SD) | 2.24 (3.69) | 2.24 (3.69) |
| - Min - Max | 0.01 - 11.60 | 0.01 - 11.60 |
| mmp9 (Relative units) |  |  |
| - (Missing) | 20 | 20 |
| - Mean (SD) | 0.04 (0.05) | 0.04 (0.05) |
| - Min - Max | 0.00 - 0.14 | 0.00 - 0.14 |
| pla2g7 (Relative units) |  |  |
| - (Missing) | 15 | 15 |
| - Mean (SD) | 1.13 (1.70) | 1.13 (1.70) |
| - Min - Max | 0.01 - 4.60 | 0.01 - 4.60 |
| mmp7 (Relative units) |  |  |
| - (Missing) | 16 | 16 |
| - Mean (SD) | 2.54 (4.22) | 2.54 (4.22) |
| - Min - Max | 0.02 - 10.43 | 0.02 - 10.43 |

**12M-post**

| **Variable** | **Non-Tol (N=28)** | **Tol (N=17)** | **Total (N=45)** | **Test** | **Statistic** | **p-value** |
| --- | --- | --- | --- | --- | --- | --- |
| TSDR-Foxp3 methylation (%) |  |  |  | t-test | t (29) = -1.393 | 0,174 |
| - N | 16 | 15 | 31 |  |  |  |
| - Mean (SD) | 92.31 (2.08) | 93.19 (1.32) | 92.73 (1.78) |  |  |  |
| - Median (Q1, Q3) | 92.39 (91.36, 94.07) | 93.20 (92.22, 93.93) | 92.79 (91.70, 94.08) |  |  |  |
| - Range | 87.08 - 95.03 | 91.21 - 95.37 | 87.08 - 95.37 |  |  |  |
| hamp_1 (Relative units) |  |  |  | Mann-Whitney | W = 143.000 | 0,572 |
| - N | 16 | 16 | 32 |  |  |  |
| - Mean (SD) | 2.79 (3.95) | 1.21 (1.31) | 2.00 (3.00) |  |  |  |
| - Median (Q1, Q3) | 1.43 (0.11, 4.03) | 0.63 (0.28, 1.71) | 0.84 (0.16, 2.65) |  |  |  |
| - Range | 0.01 - 14.17 | 0.03 - 4.43 | 0.01 - 14.17 |  |  |  |
| socs_1 (Relative units) |  |  |  | Mann-Whitney | W = 97.000 | 0,963 |
| - N | 14 | 14 | 28 |  |  |  |
| - Mean (SD) | 0.04 (0.08) | 0.07 (0.16) | 0.05 (0.12) |  |  |  |
| - Median (Q1, Q3) | 0.01 (0.00, 0.04) | 0.01 (0.00, 0.01) | 0.01 (0.00, 0.02) |  |  |  |
| - Range | 0.00 - 0.27 | 0.00 - 0.46 | 0.00 - 0.46 |  |  |  |
| tfrc_1 (Relative units) |  |  |  | Mann-Whitney | W = 134.000 | 0,58 |
| - N | 15 | 16 | 31 |  |  |  |
| - Mean (SD) | 0.10 (0.14) | 0.07 (0.08) | 0.08 (0.12) |  |  |  |
| - Median (Q1, Q3) | 0.05 (0.03, 0.08) | 0.03 (0.03, 0.07) | 0.03 (0.03, 0.07) |  |  |  |
| - Range | 0.01 - 0.53 | 0.01 - 0.32 | 0.01 - 0.53 |  |  |  |
| gbp2 (Relative units) |  |  |  | Mann-Whitney | W = 46.000 | 0,602 |
| - N | 9 | 12 | 21 |  |  |  |
| - Mean (SD) | 1.47 (2.01) | 1.32 (1.62) | 1.39 (1.75) |  |  |  |
| - Median (Q1, Q3) | 0.56 (0.04, 2.00) | 0.72 (0.45, 1.74) | 0.56 (0.34, 1.80) |  |  |  |
| - Range | 0.01 - 5.48 | 0.06 - 6.06 | 0.01 - 6.06 |  |  |  |
| gpnmb (Relative units) |  |  |  | Mann-Whitney | W = 48.000 | 1 |
| - N | 8 | 12 | 20 |  |  |  |
| - Mean (SD) | 0.21 (0.18) | 0.17 (0.20) | 0.19 (0.19) |  |  |  |
| - Median (Q1, Q3) | 0.25 (0.02, 0.36) | 0.10 (0.04, 0.21) | 0.11 (0.03, 0.35) |  |  |  |
| - Range | 0.01 - 0.41 | 0.03 - 0.64 | 0.01 - 0.64 |  |  |  |
| hla_dma (Relative units) |  |  |  | Mann-Whitney | W = 59.000 | 0,754 |
| - N | 9 | 12 | 21 |  |  |  |
| - Mean (SD) | 6.54 (5.98) | 5.04 (4.55) | 5.69 (5.12) |  |  |  |
| - Median (Q1, Q3) | 4.39 (1.77, 10.91) | 4.33 (1.95, 5.28) | 4.39 (1.90, 6.34) |  |  |  |
| - Range | 0.85 - 18.86 | 0.68 - 17.58 | 0.68 - 18.86 |  |  |  |
| hmmr (Relative units) |  |  |  | Mann-Whitney | W = 58.000 | 0,808 |
| - N | 9 | 12 | 21 |  |  |  |
| - Mean (SD) | 1.28 (2.23) | 0.51 (0.59) | 0.84 (1.53) |  |  |  |
| - Median (Q1, Q3) | 0.39 (0.04, 1.39) | 0.29 (0.08, 0.62) | 0.37 (0.07, 0.74) |  |  |  |
| - Range | 0.01 - 6.96 | 0.01 - 1.70 | 0.01 - 6.96 |  |  |  |
| mmp9 (Relative units) |  |  |  | Mann-Whitney | W = 44.000 | 0,968 |
| - N | 9 | 10 | 19 |  |  |  |
| - Mean (SD) | 0.04 (0.05) | 0.03 (0.04) | 0.03 (0.04) |  |  |  |
| - Median (Q1, Q3) | 0.01 (0.00, 0.03) | 0.01 (0.01, 0.04) | 0.01 (0.00, 0.04) |  |  |  |
| - Range | 0.00 - 0.13 | 0.00 - 0.12 | 0.00 - 0.13 |  |  |  |
| pla2g7 (Relative units) |  |  |  | Mann-Whitney | W = 68.000 | 0,345 |
| - N | 9 | 12 | 21 |  |  |  |
| - Mean (SD) | 1.43 (3.20) | 0.28 (0.48) | 0.77 (2.13) |  |  |  |
| - Median (Q1, Q3) | 0.19 (0.08, 0.75) | 0.07 (0.03, 0.29) | 0.16 (0.04, 0.36) |  |  |  |
| - Range | 0.00 - 9.85 | 0.01 - 1.69 | 0.00 - 9.85 |  |  |  |
| mmp7 (Relative units) |  |  |  | Mann-Whitney | W = 79.000 | 0,228 |
| - N | 10 | 12 | 22 |  |  |  |
| - Mean (SD) | 7.68 (16.02) | 0.41 (0.51) | 3.71 (11.13) |  |  |  |
| - Median (Q1, Q3) | 0.45 (0.26, 1.84) | 0.23 (0.09, 0.45) | 0.36 (0.11, 0.69) |  |  |  |
| - Range | 0.04 - 48.00 | 0.02 - 1.81 | 0.02 - 48.00 |  |  |  |

**Odds ratio**

| **Variable** | **Non-Tol (N=28)** | **Tol (N=17)** | **OR** | **p.ratio** |
| --- | --- | --- | --- | --- |
| TSDR-Foxp3 methylation (%) | 89.6 [87.3;90.8] | 89.3 [88.6;90.3] | 1.06 [0.83;1.35] | 0,656 |
| hamp_1 (Relative units) | 2.16 [0.80;3.78] | 3.32 [1.89;6.57] | 0.99 [0.92;1.08] | 0,879 |
| socs_1 (Relative units) | 0.04 [0.01;0.39] | 0.02 [0.01;0.47] | 0.85 [0.51;1.42] | 0,53 |
| tfrc_1 (Relative units) | 0.05 [0.03;0.12] | 0.03 [0.02;0.05] | 0.00 [0.00;39.3] | 0,196 |
| CD4+Foxp3+ cells (number) | 2.00 [0.00;5.50] | 3.00 [0.50;7.50] | 1.00 [0.92;1.10] | 0,928 |
| gbp2 (Relative units) | 1.36 [0.56;3.34] | 2.62 [0.61;6.01] | 1.00 [0.90;1.11] | 0,962 |
| gpnmb (Relative units) | 0.16 [0.06;0.95] | 0.14 [0.07;0.48] | 0.60 [0.23;1.53] | 0,285 |
| hla_dma (Relative units) | 2.01 [0.82;5.64] | 2.57 [0.97;4.75] | 1.04 [0.94;1.15] | 0,469 |
| hmmr (Relative units) | 0.97 [0.20;1.94] | 0.58 [0.19;1.18] | 0.93 [0.74;1.18] | 0,561 |
| mmp9 (Relative units) | 0.04 [0.02;0.25] | 0.02 [0.01;0.11] | 0.00 [0.00;6.81] | 0,117 |
| pla2g7 (Relative units) | 0.49 [0.12;1.45] | 0.43 [0.20;1.32] | 0.92 [0.66;1.28] | 0,623 |
| mmp7 (Relative units) | 0.48 [0.15;4.25] | 0.41 [0.12;4.63] | 0.98 [0.90;1.07] | 0,707 |
