# Supplementary material for: Longitudinal biomarker progression and validation for predicting operational tolerance in a prospective multicenter liver transplantation immunosuppression withdrawal trial
Source: PLoS One. 2025 Dec 8;20(12):e0326442. doi: 10.1371/journal.pone.0326442 (PMC12685220; doi:10.1371/journal.pone.0326442)
Supplement: S6 Table — (DOCX) [file pone.0326442.s008.docx]

**Supplementary Table 6.-** Statistical analysis of different variables evaluated longitudinally in liver tissue and whole blood along the withdrawal protocol.

**TOL Tissue**

| **Met_Ratio** |  |  |  |  |  |
| --- | --- | --- | --- | --- | --- |
| **contrast** | **Estimated mean diff. (95%CI)** | **SE** | **t.ratio** | **p.value** | **Signif** |
| Basal-12 M-post | -4.115 (-5.273, -2.957) | 0,568 | -7,239 | 0 | *** |
|  |  |  |  |  |  |
| **HAMP** |  |  |  |  |  |
| **contrast** | **Estimated mean diff. (95%CI)** | **SE** | **t.ratio** | **p.value** | **Signif** |
| Basal-12 M-post | 3.569 (1.214, 5.925) | 1,117 | 3,194 | 0,005 | ** |
|  |  |  |  |  |  |
| **GBP2** |  |  |  |  |  |
| **contrast** | **Estimated mean diff. (95%CI)** | **SE** | **t.ratio** | **p.value** | **Signif** |
| Basal-12 M-post | 3.292 (0.025, 6.559) | 1,595 | 2,064 | 0,048 | * |

**Non-TOL Tissue**

| **Met_Ratio** |  |  |  |  |  |
| --- | --- | --- | --- | --- | --- |
| **contrast** | **Estimated mean diff. (95%CI)** | **SE** | **t.ratio** | **p.value** | **Signif** |
| Basal-R | 1.535 (0.004, 3.067) | 0,747 | 2,056 | 0,049 | * |
| R - 12M-post | -2.169 (-3.874, -0.463) | 0,835 | -2,598 | 0,014 | * |
| Basal - 1M-post2 | -3.704 (-5.2, -2.208) | 0,729 | -5,081 | 0 | *** |
|  |  |  |  |  |  |
| **GPNMB** |  |  |  |  |  |
| **contrast** | **Estimated mean diff. (95%CI)** | **SE** | **t.ratio** | **p.value** | **Signif** |
| Basal-R | -0.384 (-0.936, 0.168) | 0,268 | -1,432 | 0,164 | ns |
| R - 12M-post | 0.218 (-0.454, 0.891) | 0,328 | 0,666 | 0,511 | ns |
| Basal - 1M-post2 | 0.603 (0.01, 1.195) | 0,286 | 2,103 | 0,046 | * |
|  |  |  |  |  |  |
| **MMP9** |  |  |  |  |  |
| **contrast** | **Estimated mean diff. (95%CI)** | **SE** | **t.ratio** | **p.value** | **Signif** |
| Basal-R | -0.097 (-0.19, -0.003) | 0,046 | -2,113 | 0,044 | * |
| R - 12M-post | 0 (-0.096, 0.096) | 0,047 | -0,008 | 0,994 | ns |
| Basal - 1M-post2 | 0.096 (0.005, 0.187) | 0,044 | 2,173 | 0,039 | * |

**TOL Blood**

| **SENP6** |  |  |  |  |  |
| --- | --- | --- | --- | --- | --- |
| **contrast** | **Estimated mean diff. (95%CI)** | **SE** | **t.ratio** | **p.value** | **Signif** |
| BASAL - 2M | 0.508 (-0.715, 1.73) | 0,615 | 0,825 | 0,412 | ns |
| BASAL - 6M | -0.898 (-2.102, 0.305) | 0,606 | -1,482 | 0,142 | ns |
| BASAL - TOL | -0.376 (-1.619, 0.867) | 0,626 | -0,601 | 0,549 | ns |
| BASAL - 12M_POST | -0.467 (-1.689, 0.756) | 0,615 | -0,758 | 0,45 | ns |
| BASAL - 6M_POST | 0.276 (-1.082, 1.634) | 0,684 | 0,404 | 0,687 | ns |
| 2M - 6M | -1.406 (-2.628, -0.184) | 0,615 | -2,284 | 0,025 | * |
| 2M - TOL | -0.884 (-2.145, 0.378) | 0,635 | -1,391 | 0,167 | ns |
| 2M - 12M_POST | -0.974 (-2.215, 0.266) | 0,625 | -1,56 | 0,122 | ns |
| 2M - 6M_POST | -0.232 (-1.606, 1.143) | 0,692 | -0,335 | 0,739 | ns |
| 6M - TOL | 0.522 (-0.721, 1.765) | 0,626 | 0,834 | 0,406 | ns |
| 6M - 12M_POST | 0.432 (-0.791, 1.654) | 0,615 | 0,701 | 0,485 | ns |
| 6M - 6M_POST | 1.174 (-0.184, 2.532) | 0,684 | 1,718 | 0,089 | ns |
| TOL - 12M_POST | -0.091 (-1.352, 1.171) | 0,635 | -0,143 | 0,887 | ns |
| TOL - 6M_POST | 0.652 (-0.741, 2.045) | 0,701 | 0,93 | 0,355 | ns |
| 6M_POST - 12M_POST | 0.743 (-0.632, 2.117) | 0,692 | 1,073 | 0,286 | ns |
|  |  |  |  |  |  |
| **FOXP3** |  |  |  |  |  |
| **contrast** | **Estimated mean diff. (95%CI)** | **SE** | **t.ratio** | **p.value** | **Signif** |
| BASAL - 2M | -0.001 (-0.016, 0.013) | 0,007 | -0,147 | 0,884 | ns |
| BASAL - 6M | 0.008 (-0.006, 0.022) | 0,007 | 1,097 | 0,276 | ns |
| BASAL - TOL | 0.021 (0.006, 0.035) | 0,007 | 2,831 | 0,006 | ** |
| BASAL - 12M_POST | 0.011 (-0.003, 0.026) | 0,007 | 1,55 | 0,125 | ns |
| BASAL - 6M_POST | 0.019 (0.003, 0.036) | 0,008 | 2,388 | 0,019 | * |
| 2M - 6M | 0.009 (-0.006, 0.023) | 0,007 | 1,226 | 0,224 | ns |
| 2M - TOL | 0.022 (0.007, 0.036) | 0,007 | 2,93 | 0,004 | ** |
| 2M - 12M_POST | 0.012 (-0.002, 0.027) | 0,007 | 1,669 | 0,099 | ns |
| 2M - 6M_POST | 0.02 (0.004, 0.037) | 0,008 | 2,484 | 0,015 | * |
| 6M - TOL | 0.013 (-0.002, 0.027) | 0,007 | 1,752 | 0,084 | ns |
| 6M - 12M_POST | 0.003 (-0.011, 0.018) | 0,007 | 0,471 | 0,639 | ns |
| 6M - 6M_POST | 0.012 (-0.005, 0.028) | 0,008 | 1,425 | 0,158 | ns |
| TOL - 12M_POST | -0.009 (-0.024, 0.005) | 0,007 | -1,261 | 0,211 | ns |
| TOL - 6M_POST | -0.001 (-0.018, 0.015) | 0,008 | -0,139 | 0,89 | ns |
| 6M_POST - 12M_POST | 0.008 (-0.008, 0.025) | 0,008 | 0,99 | 0,325 | ns |
|  |  |  |  |  |  |
| **IKF2** |  |  |  |  |  |
| **contrast** | **Estimated mean diff. (95%CI)** | **SE** | **t.ratio** | **p.value** | **Signif** |
| BASAL - 2M | 0.05 (-0.006, 0.106) | 0,028 | 1,78 | 0,078 | ns |
| BASAL - 6M | -0.023 (-0.078, 0.031) | 0,027 | -0,856 | 0,394 | ns |
| BASAL - TOL | 0.07 (0.014, 0.126) | 0,028 | 2,471 | 0,015 | * |
| BASAL - 12M_POST | 0.058 (0.003, 0.113) | 0,028 | 2,104 | 0,038 | * |
| BASAL - 6M_POST | 0.086 (0.025, 0.147) | 0,031 | 2,808 | 0,006 | ** |
| 2M - 6M | -0.073 (-0.129, -0.018) | 0,028 | -2,609 | 0,011 | * |
| 2M - TOL | 0.019 (-0.038, 0.077) | 0,029 | 0,671 | 0,504 | ns |
| 2M - 12M_POST | 0.008 (-0.049, 0.065) | 0,029 | 0,285 | 0,777 | ns |
| 2M - 6M_POST | 0.036 (-0.026, 0.099) | 0,032 | 1,148 | 0,254 | ns |
| 6M - TOL | 0.093 (0.037, 0.149) | 0,028 | 3,3 | 0,001 | ns |
| 6M - 12M_POST | 0.082 (0.027, 0.137) | 0,028 | 2,947 | 0,004 | ** |
| 6M - 6M_POST | 0.11 (0.049, 0.171) | 0,031 | 3,567 | 0,001 | ns |
| TOL - 12M_POST | -0.011 (-0.068, 0.045) | 0,029 | -0,397 | 0,692 | ns |
| TOL - 6M_POST | 0.017 (-0.046, 0.079) | 0,032 | 0,531 | 0,596 | ns |
| 6M_POST - 12M_POST | 0.028 (-0.034, 0.09) | 0,031 | 0,903 | 0,369 | ns |

**Non-TOL Blood**

| **Met_Ratio** |  |  |  |  |  |
| --- | --- | --- | --- | --- | --- |
| **contrast** | **Estimated mean diff. (95%CI)** | **SE** | **t.ratio** | **p.value** | **Signif** |
| BASAL - 2M | -0.055 (-0.818, 0.708) | 0,385 | -0,143 | 0,886 | ns |
| BASAL - 6M | -0.038 (-0.872, 0.796) | 0,421 | -0,09 | 0,928 | ns |
| BASAL - R | 0.937 (0.073, 1.801) | 0,436 | 2,149 | 0,034 | * |
| BASAL - 12M_POST | -0.148 (-1.012, 0.716) | 0,436 | -0,339 | 0,735 | ns |
| BASAL - 6M_POST | 0.31 (-0.59, 1.211) | 0,455 | 0,683 | 0,496 | ns |
| 2M - 6M | 0.017 (-0.817, 0.851) | 0,421 | 0,041 | 0,967 | ns |
| 2M - R | 0.992 (0.128, 1.856) | 0,436 | 2,276 | 0,025 | * |
| 2M - 12M_POST | -0.093 (-0.956, 0.771) | 0,436 | -0,212 | 0,832 | ns |
| 2M - 6M_POST | 0.366 (-0.535, 1.266) | 0,455 | 0,804 | 0,423 | ns |
| 6M - R | 0.975 (0.048, 1.902) | 0,468 | 2,084 | 0,039 | * |
| 6M - 12M_POST | -0.11 (-1.037, 0.817) | 0,468 | -0,235 | 0,815 | ns |
| 6M - 6M_POST | 0.348 (-0.613, 1.31) | 0,485 | 0,718 | 0,474 | ns |
| R - 12M_POST | -1.085 (-2.039, -0.131) | 0,482 | -2,253 | 0,026 | * |
| R - 6M_POST | -0.627 (-1.614, 0.361) | 0,498 | -1,257 | 0,211 | ns |
| 6M_POST - 12M_POST | 0.458 (-0.529, 1.446) | 0,498 | 0,92 | 0,36 | ns |
|  |  |  |  |  |  |
| **FEM1C** |  |  |  |  |  |
| **contrast** | **Estimated mean diff. (95%CI)** | **SE** | **t.ratio** | **p.value** | **Signif** |
| BASAL - 2M | 1.187 (0.245, 2.129) | 0,474 | 2,505 | 0,014 | * |
| BASAL - 6M | 1.496 (0.508, 2.484) | 0,498 | 3,007 | 0,003 | ** |
| BASAL - R | 1.551 (0.498, 2.603) | 0,53 | 2,924 | 0,004 | ** |
| BASAL - 12M_POST | 0.183 (-0.912, 1.279) | 0,552 | 0,332 | 0,74 | ns |
| BASAL - 6M_POST | 1.216 (0.142, 2.29) | 0,541 | 2,246 | 0,027 | * |
| 2M - 6M | 0.309 (-0.689, 1.306) | 0,502 | 0,615 | 0,54 | ns |
| 2M - R | 0.363 (-0.698, 1.425) | 0,535 | 0,679 | 0,499 | ns |
| 2M - 12M_POST | -1.004 (-2.107, 0.099) | 0,556 | -1,806 | 0,074 | ns |
| 2M - 6M_POST | 0.029 (-1.053, 1.111) | 0,545 | 0,053 | 0,958 | ns |
| 6M - R | 0.055 (-1.048, 1.157) | 0,556 | 0,098 | 0,922 | ns |
| 6M - 12M_POST | -1.313 (-2.455, -0.17) | 0,576 | -2,28 | 0,025 | * |
| 6M - 6M_POST | -0.28 (-1.402, 0.842) | 0,566 | -0,495 | 0,622 | ns |
| R - 12M_POST | -1.367 (-2.565, -0.169) | 0,604 | -2,264 | 0,026 | * |
| R - 6M_POST | -0.334 (-1.513, 0.844) | 0,594 | -0,563 | 0,575 | ns |
| 6M_POST - 12M_POST | 1.033 (-0.178, 2.243) | 0,609 | 1,695 | 0,094 | ns |
|  |  |  |  |  |  |
| **IKF2** |  |  |  |  |  |
| **contrast** | **Estimated mean diff. (95%CI)** | **SE** | **t.ratio** | **p.value** | **Signif** |
| BASAL - 2M | 0.003 (-0.027, 0.034) | 0,015 | 0,219 | 0,827 | ns |
| BASAL - 6M | 0.016 (-0.017, 0.05) | 0,017 | 0,976 | 0,332 | ns |
| BASAL - R | 0.032 (-0.003, 0.066) | 0,017 | 1,835 | 0,07 | ns |
| BASAL - 12M_POST | 0.035 (0, 0.07) | 0,018 | 1,989 | 0,05 | * |
| BASAL - 6M_POST | 0.031 (-0.004, 0.066) | 0,018 | 1,781 | 0,078 | ns |
| 2M - 6M | 0.013 (-0.02, 0.046) | 0,016 | 0,794 | 0,429 | ns |
| 2M - R | 0.028 (-0.005, 0.062) | 0,017 | 1,671 | 0,098 | ns |
| 2M - 12M_POST | 0.032 (-0.003, 0.066) | 0,017 | 1,822 | 0,072 | ns |
| 2M - 6M_POST | 0.028 (-0.007, 0.063) | 0,017 | 1,61 | 0,111 | ns |
| 6M - R | 0.015 (-0.021, 0.051) | 0,018 | 0,85 | 0,397 | ns |
| 6M - 12M_POST | 0.019 (-0.018, 0.056) | 0,019 | 1,004 | 0,318 | ns |
| 6M - 6M_POST | 0.015 (-0.022, 0.052) | 0,019 | 0,806 | 0,422 | ns |
| R - 12M_POST | 0.003 (-0.034, 0.041) | 0,019 | 0,171 | 0,864 | ns |
| R - 6M_POST | 0 (-0.038, 0.037) | 0,019 | -0,022 | 0,982 | ns |
| 6M_POST - 12M_POST | -0.004 (-0.041, 0.034) | 0,019 | -0,195 | 0,846 | ns |
|  |  |  |  |  |  |
| **miR31** |  |  |  |  |  |
| **contrast** | **Estimated mean diff. (95%CI)** | **SE** | **t.ratio** | **p.value** | **Signif** |
| BASAL - 2M | 2.265 (-0.733, 5.262) | 1,509 | 1,501 | 0,137 | ns |
| BASAL - 6M | 3.336 (0.26, 6.413) | 1,549 | 2,154 | 0,034 | * |
| BASAL - R | 3.139 (-0.136, 6.414) | 1,65 | 1,903 | 0,06 | ns |
| BASAL - 12M_POST | -0.376 (-3.781, 3.03) | 1,716 | -0,219 | 0,827 | ns |
| BASAL - 6M_POST | 3.235 (-0.171, 6.64) | 1,716 | 1,885 | 0,062 | ns |
| 2M - 6M | 1.071 (-2.129, 4.271) | 1,612 | 0,665 | 0,508 | ns |
| 2M - R | 0.874 (-2.522, 4.271) | 1,712 | 0,511 | 0,611 | ns |
| 2M - 12M_POST | -2.64 (-6.152, 0.871) | 1,77 | -1,492 | 0,139 | ns |
| 2M - 6M_POST | 0.97 (-2.549, 4.489) | 1,774 | 0,547 | 0,586 | ns |
| 6M - R | -0.197 (-3.647, 3.253) | 1,738 | -0,113 | 0,91 | ns |
| 6M - 12M_POST | -3.712 (-7.28, -0.143) | 1,798 | -2,064 | 0,042 | * |
| 6M - 6M_POST | -0.102 (-3.67, 3.467) | 1,798 | -0,056 | 0,955 | ns |
| R - 12M_POST | -3.515 (-7.251, 0.221) | 1,883 | -1,867 | 0,065 | ns |
| R - 6M_POST | 0.095 (-3.64, 3.831) | 1,882 | 0,051 | 0,96 | ns |
| 6M_POST - 12M_POST | 3.61 (-0.197, 7.418) | 1,916 | 1,884 | 0,063 | ns |
