# Supplementary material for: Longitudinal biomarker progression and validation for predicting operational tolerance in a prospective multicenter liver transplantation immunosuppression withdrawal trial
Source: PLoS One. 2025 Dec 8;20(12):e0326442. doi: 10.1371/journal.pone.0326442 (PMC12685220; doi:10.1371/journal.pone.0326442)
Supplement: S8 Table — (DOCX) [file pone.0326442.s010.docx]

**Supplementary Table 8.-** Statistics from linear mixed model with flexible effects of different variables evaluated longitudinally in liver tissue and whole blood along the withdrawal protocol.

**Tissue**

| **Met_Ratio** |  |  |  |  |  |
| --- | --- | --- | --- | --- | --- |
| **Contrast** | **edf** | **Ref.df** | **F.statistic** | **P.value** | **Signification** |
| s(Time):non-Tol Group | 1,001 | 1,001 | 24,517 | 0 | *** |
| s(Time):Tol Group | 1,001 | 1,002 | 26,426 | 0 | *** |
| s(id) | 17,586 | 42 | 0,717 | 0,026 | * |
|  |  |  |  |  |  |
| **HAMP** |  |  |  |  |  |
| **Contrast** | **edf** | **Ref.df** | **F.statistic** | **P.value** | **Signification** |
| s(Time):non-Tol Group | 1 | 1 | 0,035 | 0,852 | ns |
| s(Time):Tol Group | 1 | 1 | 4,808 | 0,032 | * |
| s(id) | 20,208 | 42 | 0,801 | 0,033 | * |
|  |  |  |  |  |  |
| **GBP2** |  |  |  |  |  |
| **Contrast** | **edf** | **Ref.df** | **F.statistic** | **P.value** | **Signification** |
| s(Time):non-Tol Group | 1 | 1 | 4,482 | 0,038 | * |
| s(Time):Tol Group | 1,001 | 1,001 | 2,718 | 0,104 | ns |
| s(id) | 0,931 | 37 | 0,026 | 0,446 | ns |
|  |  |  |  |  |  |
| **GPNMB** |  |  |  |  |  |
| **Contrast** | **edf** | **Ref.df** | **F.statistic** | **P.value** | **Signification** |
| s(Time):non-Tol Group | 1 | 1 | 6,102 | 0,017 | * |
| s(Time):Tol Group | 1 | 1 | 1,27 | 0,265 | ns |
| s(id) | 11,714 | 37 | 0,497 | 0,075 | ns |

**Blood**

| **FOXP3** |  |  |  |  |  |
| --- | --- | --- | --- | --- | --- |
| **Contrast** | **edf** | **Ref.df** | **F.statistic** | **P.value** | **Signification** |
| s(Time):non-Tol Group | 1 | 1,001 | 0,198 | 0,657 | ns |
| s(Time):Tol Group | 2,424 | 3,014 | 3,254 | 0,022 | * |
| s(id) | 0,933 | 42 | 0,023 | 0,438 | ns |
|  |  |  |  |  |  |
| **IKF2** |  |  |  |  |  |
| **Contrast** | **edf** | **Ref.df** | **F.statistic** | **P.value** | **Signification** |
| s(Time):non-Tol Group | 1,466 | 1,791 | 1,433 | 0,168 | ns |
| s(Time):Tol Group | 3,728 | 4,586 | 3,823 | 0,004 | ** |
| s(id) | 13,737 | 42 | 0,489 | 0,039 | * |
