# Supplementary material for: Longitudinal biomarker progression and validation for predicting operational tolerance in a prospective multicenter liver transplantation immunosuppression withdrawal trial
Source: PLoS One. 2025 Dec 8;20(12):e0326442. doi: 10.1371/journal.pone.0326442 (PMC12685220; doi:10.1371/journal.pone.0326442)
Supplement: Attachment — Submitted filename: Response to Reviewers.docx [file pone.0326442.s012.docx]

**José Antonio Pons, PhD, MD**

Head of area

*Hepatology and Liver Transplant Unit*

*University Clinical Hospital Virgen de la Arrixaca*

*Murcia's Biohealth Research Institute-Virgen de la Arrixaca*

*30120 Murcia, Spain*

*E-mail:* [*joseapons.imib.arrixaca@gmail.com*](mailto:joseapons.imib.arrixaca@gmail.com)

**Manuscript Reference PONE-D-25-05354**

Point-by-point Rebuttal Letter

We especially thank the reviewers and editors for their comments. We have revised the manuscript in accordance with their suggestions and we believe these revisions will satisfy the issues raised by the referees. Changes in the revised manuscript are displayed as "tracked changes". Also, a clean version of the manuscript has been submitted.

**Journal Requirements:**

1. When submitting your revision, we need you to address these additional requirements.

Please ensure that your manuscript meets PLOS ONE's style requirements, including those for file naming.

*We have revised the manuscript in accordance with all PLOS ONE style requirements.*

2. Thank you for stating the following financial disclosure:

J.A.P. was founded by Instituto de Salud Carlos III (PI17/00489; PI23/00013), co-funded by the European Union. J.I.H. was founded by Instituto de Salud Carlos III (PI17/00699), co-funded by the European Union. A.B-M. was funded by Instituto de Salud Carlos III (PI20/00185; DTS23/00013; PI24/00129), co-funded by the European Union.

Please state what role the funders took in the study. If the funders had no role, please state: "The funders had no role in study design, data collection and analysis, decision to publish, or preparation of the manuscript."

If this statement is not correct you must amend it as needed.

Please include this amended Role of Funder statement in your cover letter; we will change the online submission form on your behalf.

*We have incorporated the updated Role of Funder statement in the cover letter, as requested.*

3. Thank you for stating the following in the Acknowledgments Section of your manuscript:

This work was supported by the Instituto de Salud Carlos III (ISCIII) Platform for Clinical Research Support - SCReN (Spanish Clinical Research Network), funded by ISCIII through project PI17/00489 and co-funded by the European Regional Development Fund (ERDF) “A way of making Europe.” It also received support from the Clinical Trials Platform of the Instituto Murciano de Investigación Biosanitaria (IMIB-Pascual Parrilla). We are particularly grateful for the generous contribution of the patients and the collaboration of Biobank Network of the Region of Murcia, BIOBANC-MUR, registered on the Registro Nacional de Biobancos with registration number B.0000859. BIOBANC-MUR and supported by the ISC III (PT20/00109), by “IMIB-Pascual Parrilla” and by “Consejeria de Salud de la Comunidad Autónoma de la Región de Murcia”. Likewise, the authors thank the Genomics and Pathology Core at the IMIB-Pascual Parrilla for support.

We note that you have provided funding information that is not currently declared in your Funding Statement. However, funding information should not appear in the Acknowledgments section or other areas of your manuscript. We will only publish funding information present in the Funding Statement section of the online submission form.

Please remove any funding-related text from the manuscript and let us know how you would like to update your Funding Statement. Currently, your Funding Statement reads as follows:

J.A.P. was founded by Instituto de Salud Carlos III (PI17/00489; PI23/00013), co-funded by the European Union. J.I.H. was founded by Instituto de Salud Carlos III (PI17/00699), co-funded by the European Union. A.B-M. was funded by Instituto de Salud Carlos III (PI20/00185; DTS23/00013; PI24/00129), co-funded by the European Union.

Please include your amended statements within your cover letter; we will change the online submission form on your behalf.

*In the revised manuscript, we have ensured that the Acknowledgements section no longer contains any funding information.*

4. In the online submission form, you indicated that “The data that support the findings of this study are available from the corresponding author upon reasonable request. Some data may not be made available because of privacy or ethical restrictions.”

All PLOS journals now require all data underlying the findings described in their manuscript to be freely available to other researchers, either a. In a public repository, b. Within the manuscript itself, or c. Uploaded as supplementary information.

This policy applies to all data except where public deposition would breach compliance with the protocol approved by your research ethics board. If your data cannot be made publicly available for ethical or legal reasons (e.g., public availability would compromise patient privacy), please explain your reasons on resubmission and your exemption request will be escalated for approval.

*We apologize for the confusion. Please note that all relevant data have been included in the main manuscript and/or the supplementary files.*

**Additional Editor Comments:**

Please shrink your manuscript, add the clinical applicability and double check your references.

*We sincerely thank the Editor for the additional comments and constructive guidance. In response, we have carefully revised the manuscript as follows:*

*Manuscript length: We have carefully reduced the length of the text, focusing on improving clarity and eliminating redundancy, particularly in the Discussion and Figure Legends, while preserving the scientific integrity and core findings.*

*Clinical applicability: We have expanded the discussion and conclusions to better articulate the clinical relevance of our findings. In particular, we included a dedicated paragraph outlining the potential for real-world implementation, cost considerations, and accessibility of the biomarker assays. We also responded to reviewer concerns about long-term follow-up and post-weaning clinical outcomes to contextualize the results.*

*References: All citations have been thoroughly reviewed and revised for completeness and accuracy. Where applicable, we have included DOI and PMID identifiers to align with PLOS ONE’s formatting requirements.*

*We have also addressed all reviewer comments point-by-point and incorporated the requested revisions into the main manuscript. We hope these changes meet the journal’s standards and expectations.*

*Thank you for considering our revised submission.*

**Reviewer #1:**

Dear authors,

Your manuscript is very interesting about a clinical crucial topic in liver transplantation

I have very few concerns I included only comments to improve your manuscript

I would like to know the costs of analysis and if could be done in a no-experimental setting to know if your nice study could be available to any LT Team

*We appreciate the reviewer’s interest in the real-world applicability of our findings. As suggested, we have expanded the Discussion to address this point explicitly. We now clarify that the molecular techniques used (pyrosequencing and RT-qPCR) are compatible with most hospital or reference laboratories and rely on commercially available kits. The per-sample cost is moderate and scalable, making implementation feasible in non-experimental clinical settings. These additions can be found in the final paragraph of the Discussion section, starting with “From a clinical perspective...”.*

I think the figures text are too long please try to short it

*We thank the reviewer for this suggestion. In response, we have carefully revised and shortened the figure legends throughout the manuscript, particularly Figures 2–5, to enhance clarity and conciseness while preserving essential information. We believe the revised legends now better align with the journal’s standards and improve overall readability.*

Discussion is nice but I think that is also too long please try to make it short

*We thank the reviewer for this constructive comment. In response, we have carefully revised the Discussion section and reduced its length by approximately 15–20%, while ensuring that the scientific content, clarity, and references were preserved. We streamlined the text by condensing repetitive or overly detailed sections, merging related ideas, and simplifying complex phrasing where possible. We hope the revised version better meets the journal’s expectations for conciseness and enhances overall readability.*

You mentioned limitations but include also strenghts.

*We thank the reviewer for this valuable suggestion. In response, we have now included a dedicated paragraph at the end of the Discussion section that highlights the strengths of our study. In particular, we emphasize its prospective multicenter design, the longitudinal and dual-compartment (blood and tissue) biomarker analysis, and the validation of previously published biomarkers in an independent multicenter cohort. These features support the robustness and translational value of our findings. Please see the revised manuscript, Discussion section, paragraph starting with “Despite these limitations...”.*

**Reviewer #2:**

This study is very interesting and sheds light on future research lines to predict OT in liver transplantation. There are some points to highlight to consider this study for publication:

From the 17 patients who achieved tolerance with normal liver function over one year, it would be interesting to know how many keep tolerant on the long run and how many had a rejection episode since the first year. Consider adding this clinical information.

*We thank the reviewer for this important question. None of the 17 patients who achieved operational tolerance at one year have required reintroduction of immunosuppression during the follow-up period. Two patients experienced isolated, mild elevations in liver enzymes (GPT levels of 70 and 50 IU/L, respectively) at 18 and 24 months post-IS withdrawal. These episodes resolved spontaneously within one month without treatment and were not accompanied by clinical symptoms or histological confirmation of rejection. These transient abnormalities are not uncommon in patients off IS and may reflect minor, self-limited inflammatory episodes rather than true rejection. Given the limited follow-up time and the absence of significant clinical events, all 17 patients remain off IS and continue to be monitored regularly. We have added this information to the discussion section of the Manuscript (see page 19, lines 373-376: “Notably, none of the TOL patients required reintroduction of IS during extended follow-up. Only two patients experienced mild, transient elevations in liver enzymes, which resolved spontaneously within one month, likely reflecting minor, self-limiting inflammatory responses rather than true rejection episodes [24]”).*

In the tolerance group, have you seen biomarker differences in patients with Mycophenolate mofetil on monotherapy from patients with tacrolimus based regimen. Consider adding this information if has been analyzed.

*We appreciate the reviewer’s insightful suggestion. In our cohort, only three patients within the tolerance group were receiving monotherapy with MMF, while the vast majority were on a tacrolimus-based regimen. Given the very limited number of MMF monotherapy cases, we believe that a comparative statistical analysis would lack sufficient power and could lead to misleading interpretations. For this reason, we have opted not to include subgroup comparisons based on baseline immunosuppressive regimen. However, we agree that future studies with larger sample sizes could address this interesting question more robustly.*

As mentioned in limitations, which regularization methods have been used to counteract small sample size? Please mention them If applicable.

*We thank the reviewer for pointing out the need to clarify this methodological aspect. As noted in the Methods section and detailed in the Supplementary Methods, we applied LASSO (Least Absolute Shrinkage and Selection Operator) regularization to reduce model overfitting and handle high-dimensional data in the context of our limited sample size. LASSO was used during the predictive model development phase to perform variable selection and penalize less informative features, thus ensuring stable and interpretable models. We have now explicitly mentioned this in the Manuscript to improve clarity (Discussion, page 22, lines 440-443: “However, the use of regularization methods,* ***such as the LASSO approach*** *[18], have been shown to be robust under high-dimensional settings with limited sample size, allowing for low-biased parameter estimates and effective feature selection [54]”).*

Some references are incomplete (23, 27) (missing page numbers).

*We appreciate the reviewer’s careful assessment of the reference list. Following PLOS ONE's guidelines and to ensure consistency and completeness, we have revised all references in the manuscript. Specifically, we have included both the DOI for all articles and the PMID where applicable, in accordance with the journal’s format requirements. This approach ensures persistent and unambiguous access to all cited sources, even when page numbers are not available. We have double-checked the full reference list to ensure compliance with these standards.*

Yours faithfully,

**Dr. José Antonio Pons**, on behalf of all authors.
