# Supplementary material for: Longitudinal biomarker progression and validation for predicting operational tolerance in a prospective multicenter liver transplantation immunosuppression withdrawal trial
Source: PLoS One. 2025 Dec 8;20(12):e0326442. doi: 10.1371/journal.pone.0326442 (PMC12685220; doi:10.1371/journal.pone.0326442)
Supplement: Attachment — Submitted filename: Response to Reviewers_PONE-D-25-05354R1.docx [file pone.0326442.s013.docx]

**José Antonio Pons, PhD, MD**

Head of area

*Hepatology and Liver Transplant Unit*

*University Clinical Hospital Virgen de la Arrixaca*

*Murcia's Biohealth Research Institute-Virgen de la Arrixaca*

*30120 Murcia, Spain*

*E-mail:* [*joseapons.imib.arrixaca@gmail.com*](mailto:joseapons.imib.arrixaca@gmail.com)

**Manuscript Reference PONE-D-25-05354R1**

Point-by-point Rebuttal Letter

Dear Editor and Reviewers,

We sincerely thank Reviewers 1 and 2 for their constructive feedback and for accepting our responses and revisions to the previous comments raised. We are now addressing the questions and concerns expressed by the new Reviewer 3, as indicated by the Editor. Below, we provide a point-by-point response to each of the reviewer’s remarks, incorporating clarifications and modifications to the manuscript where applicable. Changes in the revised manuscript are displayed as "tracked changes". Also, a clean version of the manuscript has been submitted.

**Journal Requirements:**

Please review your reference list to ensure that it is complete and correct. If you have cited papers that have been retracted, please include the rationale for doing so in the manuscript text, or remove these references and replace them with relevant current references. Any changes to the reference list should be mentioned in the rebuttal letter that accompanies your revised manuscript. If you need to cite a retracted article, indicate the article’s retracted status in the References list and also include a citation and full reference for the retraction notice.

*We have carefully reviewed the entire reference list to ensure its accuracy and completeness. All references are up-to-date and relevant to the study’s context.*

**Reviewer #3:**

My comments are listed below.

Line 213-215, TOL group had a longer time from transplant to IS weaning compared to non-TOL group, although it did not reach statistical significance (121.1 ± 58.3 and 92.5± 51.7 months, respectively; p = 0.094), such obvious difference in length of using IS may have influence on the outcome. Such a factor may be included in the list of candidate predictors for the predictive models.

*We sincerely appreciate the reviewer’s attention to this detail. We would like to clarify that "time from transplant to weaning" was indeed included as a candidate predictor in the LASSO model from the outset, as stated in the "Identification of potential predictive variables for operational tolerance" section (first paragraph):*

*"Starting with a model comprised of 19 biomarkers measured at baseline, along with the demographic and clinical variables such as age, sex,* ***time from transplant to weaning****, and tacrolimus concentration, the LASSO method finally selected 11 predictors...".*

*We thank the reviewer for prompting us to highlight this point.*

A total of 11 predictors were selected by LASSO, how reliable would these selected predictors be with the very limited sample size of 45 (17 TOL vs. 28 non-TOL)? The model would be overfitted by including 11 predictors in a model with n=45. To avoid overfitting and remove less impactful predictors, you may use a larger lambda as minimum lambda + 1SE. The final model would have less concern of overfitting with 3-4 selected predictors based on n=45.

*We thank the reviewer for the valuable suggestion to consider the 1-SE (1SE) rule to select a more parsimonious model with fewer predictors while maintaining cross-validated error within one standard error of the minimum rule (λmin), to reduce overfitting. In response, we fit a LASSO logistic regression model using 10-fold cross-validation and compared the models obtained via λmin and 1SE.*

*The model selected using λmin (λ = 0.86) included 11 predictors, with strong performance (Balanced Accuracy: 0.858, AUC: 0.87 [95% CI: 0.76–0.98], Kappa: 0.716). As suggested, we also evaluated the model at 1SE (λ = 0.98), which included only 4 predictors. This simpler model showed a notable reduction in classification performance (Balanced Accuracy: 0.686, AUC: 0.725 [95% CI: 0.675–0.77], Kappa: 0.337), despite retaining the biomarker of primary interest, and maintains cross-validated error within one standard error of the minimum.*

*While the 1SE model offers increased simplicity and lower overfitting risk, the λmin model demonstrated superior predictive accuracy and discriminative ability. We therefore report both models, emphasizing that the biomarker of interest remains selected in the reduced model, but highlighting the trade-off between model sparsity and predictive performance.*

*We have added these comparisons, metrics, and model details to the revised main manuscript [in Results, Methods and Discussion], and included a figure showing cross-validated error across the lambda path (Supplementary Methods and Supplementary Figure 4).*

*As suggested, we evaluated a more parsimonious model using the 1-SE rule, which selected λ = 0.98. This model includes 4 predictors — including our primary biomarker of interest — and maintains cross-validated error within one standard error of the minimum (λ = 0.86). Although the simplified model shows slightly lower classification performance (e.g., AUC = 0.725 vs. 0.87), it offers a favorable trade-off in terms of model simplicity and potential for generalization, in line with the reviewer’s concerns about overfitting.*

*To address the concern regarding overfitting, we evaluated model stability and generalizability using multiple complementary approaches:*

***(1)	Cross-Validation Error Variance:*** *We conducted 10-fold cross-validation, and the cross-validated error at the optimal lambda (λ = 0.86) was 1.093 ± 0.02. The low standard deviation (~1.8% of the mean) suggests the model performance is consistent across folds, indicating low variance and a stable model.*

***(2)	Performance Gap between Training and Validation AUC:*** *We also estimated learning curves to assess generalization. The AUC on the training data was 0.84, while the AUC on the held-out validation data was 0.76, yielding a gap of 0.08. This gap is modest and within acceptable limits in biomedical classification contexts, suggesting that overfitting is minimal and that the model generalizes reasonably well to unseen data.*

The predictive capacity and performance of the model with selected predictors should be examined and reported

*We thank the Reviewer for this important suggestion. In response, we have now explicitly reported the predictive capacity of the LASSO logistic regression models using multiple relevant metrics: the Area Under the Curve (AUC) with 95% confidence intervals, as well as sensitivity, specificity, positive predictive value, and negative predictive value.*

*To assess overall model performance, we additionally report the overall error rate, balanced accuracy, and Cohen’s kappa coefficient. These metrics are now summarized in the revised Table 2, allowing for a comprehensive evaluation of both the full model (λmin) and the reduced, more parsimonious model (λ1SE).*

Yours faithfully,

**Dr. José Antonio Pons**, on behalf of all authors.
