# Supplementary material for: Longitudinal biomarker progression and validation for predicting operational tolerance in a prospective multicenter liver transplantation immunosuppression withdrawal trial
Source: PLoS One. 2025 Dec 8;20(12):e0326442. doi: 10.1371/journal.pone.0326442 (PMC12685220; doi:10.1371/journal.pone.0326442)
Supplement: Attachment — Submitted filename: Response to Reviewers_PONE-D-25-05354R2.docx [file pone.0326442.s014.docx]

**José Antonio Pons, PhD, MD**

Head of area

*Hepatology and Liver Transplant Unit*

*University Clinical Hospital Virgen de la Arrixaca*

*Murcia's Biohealth Research Institute-Virgen de la Arrixaca*

*30120 Murcia, Spain*

*E-mail:* [*joseapons.imib.arrixaca@gmail.com*](mailto:joseapons.imib.arrixaca@gmail.com)

**Manuscript Reference PONE-D-25-05354R2**

Point-by-point Rebuttal Letter

Dear Editor and Reviewers,

We sincerely thank Reviewer 2 for their constructive feedback and for accepting our responses and revisions to the previous comments raised. We are now addressing the last concern expressed by the Reviewer 3, as indicated by the Editor. Below, we provide a point-by-point response to the reviewer’s remark, incorporating clarifications and modifications to the manuscript where applicable. Changes in the revised manuscript are displayed as "tracked changes". Also, a clean version of the manuscript has been submitted.

**Reviewer #3:**

Table 2, the numbers in the classification performance metrics should be shown in 0-100% or 0-1 decimal.

*We thank the reviewer for this valuable observation. The values in Table 2 have now been corrected and are presented in decimal format (0–1), as suggested.*

Yours faithfully,

**Dr. José Antonio Pons**, on behalf of all authors.
